# Critical Role of IL1R2‐ENO1 Interaction in Inhibiting Glycolysis‐Mediated Pyroptosis for Protection Against Lethal Sepsis

**DOI:** 10.1002/advs.202502297

**Published:** 2025-07-24

**Authors:** Chuyi Tan, Han Ma, Jespar Chen, Gaifeng Ma, Alok Jha, Sipin Tan, Yaxi Zhu, Meidong Liu, Ke Liu, Xianzhong Xiao, Monowar Aziz, Huan Chen, Ping Wang, Huali Zhang

**Affiliations:** ^1^ Center for Immunology and Inflammation The Feinstein Institutes for Medical Research 350 Community Drive Manhasset NY 11030 USA; ^2^ Key Laboratory of Sepsis Translational Medicine of Hunan, Department of Pathophysiology, School of Basic Medicine Science Central South University Changsha Hunan 410013 China; ^3^ National Medicine Functional Experimental Teaching Center Central South University Changsha Hunan 410013 China; ^4^ Departments of Surgery and Molecular Medicine Zucker School of Medicine at Hofstra/Northwell Manhasset NY 11549 USA

**Keywords:** IL1R2, enolase 1, glycolysis, macrophages, pyroptosis, sepsis

## Abstract

Immune cell metabolic reprogramming toward glycolysis is vital for sepsis defense. While interleukin 1 receptor 2 (IL1R2) acts as a decoy receptor for IL1α/β, its potential impact on cell metabolism and death during sepsis remains unclear. This study observed elevated plasma soluble IL1R2 (sIL1R2) levels in septic patients and mice. In pyroptotic macrophages, reduced intracellular IL1R2 expression led to its release extracellularly. Proteomic screening identified enolase 1 (ENO1), a key glycolysis enzyme, as the binding partner of IL1R2 in macrophages. IL1R2 suppresses ENO1 activity to inhibit glycolysis, gasdermin D (GSDMD)‐mediated pyroptosis, and inflammation in macrophages. IL1R2‐deficient mice exhibited heightened susceptibility to sepsis, with increased inflammation, organ injury, and mortality. Notably, ENO1 inhibition reduced inflammation, organ injury, and improved survival rates in septic mice. The study reveals that IL1R2 interacts with ENO1 to inhibit glycolysis‐mediated pyroptosis and inflammation in sepsis, suggesting the IL1R2‐ENO1 interaction as a promising therapeutic target of sepsis.

## Graphical abstract

The authors have discovered that intracellular interleukin 1 receptor 2 (IL1R2) negatively regulates pyroptosis and inflammation by inhibiting glycolysis in sepsis. Soluble IL1R2 is released from macrophages undergoing pyroptosis. IL1R2 acts as a novel negative regulator of glycolysis by interacting with enolase 1 (ENO1), thereby inhibiting gasdermin D (GSDMD)‐mediated pyroptosis and inflammation. The inhibition of ENO1 suppressed pyroptosis by downregulating caspase‐1.

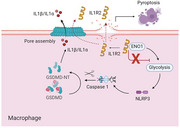

## 1 Introduction

Sepsis is defined as a life‐threatening organ dysfunction resulting from dysregulated immune responses to an infection.^[^ ^1^ ^]^ An imbalance between pro‐ and anti‐inflammatory responses within the host immune system is a common feature of sepsis. This leads to immune system overactivation and excessive cytokine production. In addition, stressed cells release alarmins, which are known as damage‐associated molecular patterns (DAMPs), and which have been demonstrated to aggravate inflammation, contribute to tissue injury, and increase mortality.^[^ ^2^ ^]^ IL1, a critical mediator of inflammation associated with high morbidity and mortality in sepsis, exists in two distinct forms: IL1α and IL1β.^[^ ^3^ ^]^ The typical mechanism for the release of IL1α/β is the activation of the inflammasome and subsequent pyroptosis. During the inflammatory response, both pathogen‐associated molecular patterns (PAMPs) and DAMPs initiate the activation of both the canonical and non‐canonical inflammasome pathways. This activation results in the cleavage of gasdermin D (GSDMD), generating its active N‐terminal fragment (N‐GSDMD), which oligomerizes in the cell membrane and forms membrane pores.^[^ ^4^ ^]^ The formation of membrane pores is followed by pyroptosis, a form of cell rupture that results in the rapid release of IL1α and IL1β into the extracellular space, thereby driving inflammation.^[^ ^5^ ^]^ Despite the pivotal role of IL1 in modulating the immune system, multicenter randomized controlled trials targeting IL1 inhibition in sepsis have not yielded favorable outcomes.^[^ ^6^ ^]^ This highlights the necessity for a more comprehensive grasp of the precise regulation of IL1 and pyroptosis, which offers considerable promise for the development of efficacious treatments for inflammatory and infectious diseases.

Decoy receptors have emerged as an evolutionarily conserved strategy to limit cytokine activity.^[^ ^7^ ^]^ One key mechanism regulating IL1 activity is the expression of IL1 receptor 2 (IL1R2) on immune cells.^[^ ^8^ ^]^ IL1R2 is selectively expressed in monocytes/macrophages, neutrophils, and B cells.^[^ ^9^ ^]^ As a decoy receptor, IL1R2 is capable of independently binding both IL1α and IL1β; however, it is unable to initiate downstream signaling due to the absence of an intracellular Toll/Interleukin‐1 receptor (TIR) domain.^[^ ^10^ ^]^ Indeed, IL1R2 acts as a competitive inhibitor of IL1‐mediated signaling, thereby dampening inflammatory responses in immune cells.^[^ ^10^, ^11^ ^]^ While previous studies focused on IL1R2 as part of the endogenous IL1 antagonist system, IL1R2 is a distinct molecule with likely more complex functions. Future research is needed to fully elucidate its precise role and potential therapeutic applications in inflammatory diseases.

Recent research has highlighted the critical role of metabolic reprogramming in immune cells and its impact on inflammatory processes and organ injury in sepsis.^[^ ^12^ ^]^ During the hyperinflammatory stage of sepsis, there is a shift in immune cell metabolism toward aerobic glycolysis, also known as the Warburg effect, with a concurrent reduction in oxidative phosphorylation (OXPHOS) despite adequate oxygen availability.^[^ ^13^ ^]^ It has been demonstrated that glycolysis represents a pivotal regulatory mechanism for inflammasome activation and IL1 production in macrophages.^[^ ^9^, ^13^, ^14^ ^]^ For instance, pyruvate kinase M2 (PKM2), a key enzyme in glycolysis, has been revealed to facilitate inflammasome activation and subsequently induce IL1β release in lipopolysaccharide (LPS)‐stimulated macrophages.^[^ ^13^, ^14^ ^]^ Furthermore, the pharmacological inhibition of glycolysis has been demonstrated to reduce inflammasome activation and IL1 production, thereby conferring protection against lethal sepsis.^[^ ^13^, ^15^ ^]^ This suggests that the inhibition of glycolysis may represent a potential therapeutic strategy for sepsis. Enolase 1 (ENO1), a glycolytic enzyme that catalyzes the conversion of 2‐phosphoglycerate to phosphoenolpyruvate, has been linked to the pathogenesis of inflammatory disorders.^[^ ^16^ ^]^ However, it remains unclear whether ENO1‐dependent glycolysis plays a regulatory role in pyroptosis in macrophages. In addition, the potential role of ENO1 in sepsis has yet to be investigated.

In this study, we demonstrate that plasma soluble IL1R2 (sIL1R2) is elevated in patients with sepsis, and that IL1R2‐deficient mice exhibited decreased survival and increased inflammation in sepsis. Importantly, we present the novel observation that IL1R2 exerts an inhibitory effect on glycolysis‐mediated pyroptosis and inflammation by interacting with ENO1 in macrophages. The pharmacological and genetic inhibition of ENO1 was observed to decrease pyroptosis‐mediated inflammation and to confer protection against sepsis. Our findings reveal a novel mechanism of the critical role of the IL1R2‐ENO1 pathway in regulating inflammation through glycolysis in sepsis. Furthermore, our results suggest that the IL1R2‐ENO1 interaction could serve as a promising therapeutic target for sepsis therapy.

## 2 Results

### 2.1 Soluble IL1R2 is released from Pyroptotic Macrophages During Sepsis

**Figure 1:**
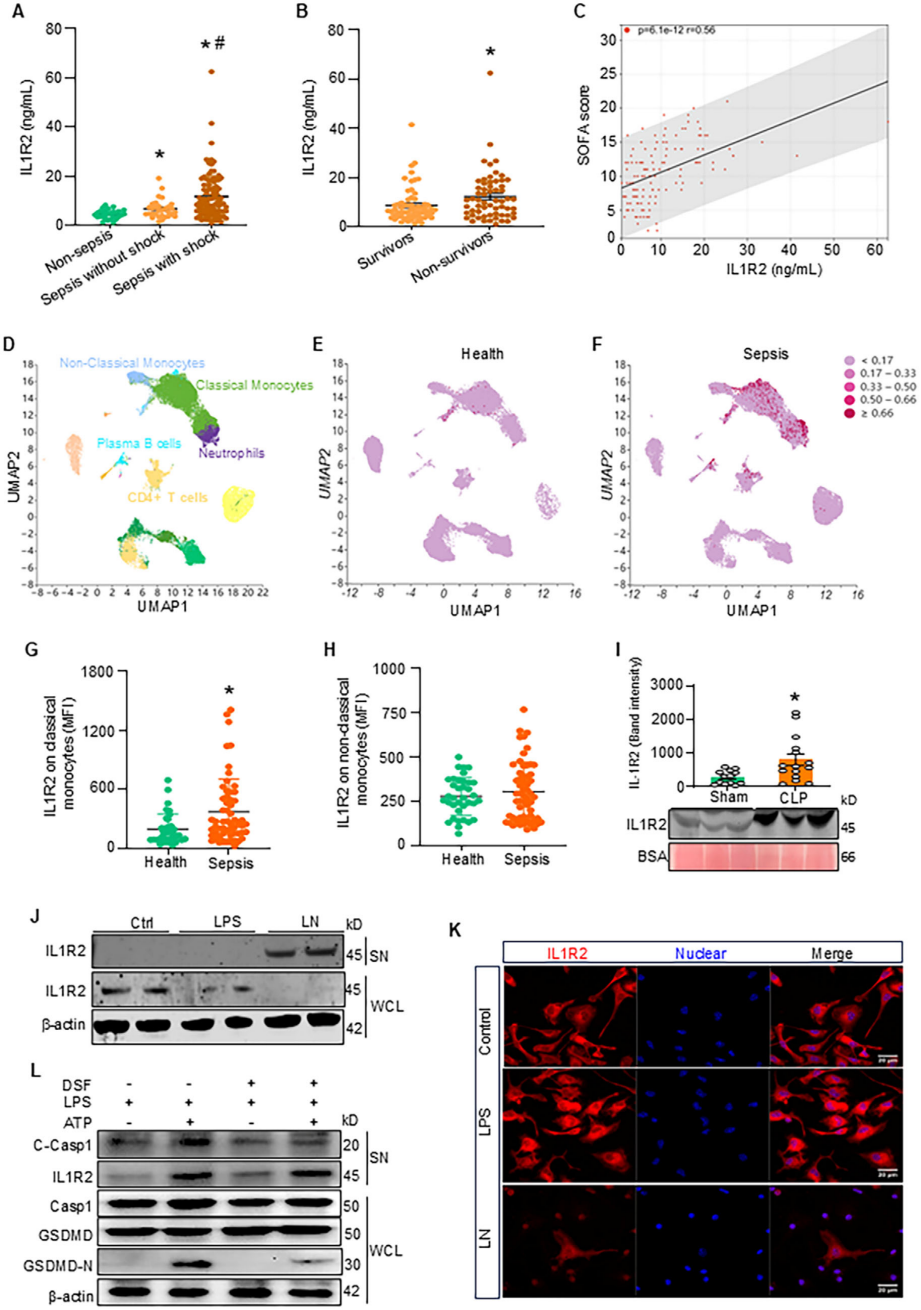
Soluble IL1R2(sIL1R2) is released from pyroptotic macrophages during sepsis. A) Plasma levels of sIL1R2 in patients without sepsis (non‐sepsis), as well as in patients with sepsis with or without shock. Data are expressed as mean ± SEM and compared by one‐way ANOVA and Tukey's multiple comparisons test. *p < 0.05 versus non‐sepsis, ^#^ *p* < 0.05 versus sepsis without shock. B) Plasma levels of sIL1R2 in septic patients, comparing survivors with non‐survivors. Data are expressed as mean ± SEM and compared by Student's t test (unpaired). *p < 0.05 versus survivor. C) The correlation analysis of plasma sIL1R2 levels and SOFA score in septic patients. D) Single‐cell RNA sequencing (scRNA‐seq) data from blood immune cells of septic patients were analyzed and visualized using uniform manifold approximation and projection (UMAP) plots, with colors in parentheses indicating the identified cell clusters. E, F) UMAP representation of snRNA‐seq data from blood immune cells of health control (E) and septic patients (F), colored according to *IL1R2* expression. G, H) The expression of IL1R2 on classical and non‐classical monocytes from health control and septic patients was detected by flow cytometry. Data are expressed as mean ± SEM and compared by Student's t test (unpaired). *p < 0.05 versus health. MFI, mean fluorescence intensity. I) The plasma levels of sIL1R2 were quantified by Western blotting in sham and cecal ligation and puncture (CLP) induced septic mice. Data are expressed as mean ± SEM and compared by Student's t test (unpaired). *p < 0.05 versus Sham. J, K) Peritoneal cavity (PerC) macrophages treated with or without LPS (1 µg mL^−1^) for 3 h, followed by nigericin (5 µM) for 1 h. LN, LPS+ nigericin. (J) The levels of IL1R2 in the whole cell lysis (WCL)and culture supernatants (SN) of these macrophages were measured. Representative images from three independent experiments with similar results. (K) The treated macrophages were fixed and stained with anti‐IL1R2 Ab (red) and DNA (Hoechst33342, blue), with images were captured by confocal microscopy. Scale bar: 20 µm. Original magnification: 630×. L) Immortalized murine bone marrow‐derived macrophages (iBMDMs) were pre‐treated with disulfiram (5 µg mL^−1^) and LPS (1 µg mL^−1^) for 3 h, then followed with ATP (5 mM) for 0.5 h. The expressions of cleaved‐Caspase1(C‐Casp1) and IL1R2 in the supernatants, and the expression of casp1, GSDMD, and N‐GSDMD in the whole cell lysis of these iBMDMs were measured. Representative images from three independent experiments with similar results.

We found that the plasma concentration of sIL1R2 was significantly increased in septic patients compared to non‐septic patients (**Figure** **1**A). Among the sub‐groups, patients with septic shock exhibited significantly higher plasma levels of sIL1R2 compared to septic patients without shock (Figure 1A). Additionally, the non‐survivor septic patients had significantly elevated plasma sIL1R2 levels compared to survivors (Figure 1B). A correlation analysis revealed a strong positive correlation between sIL1R2 levels and the SOFA score (Figure 1C). To elucidate the source of serum sIL1R2 in septic patients, we analyzed a publicly available human single‐cell RNA sequencing (scRNA‐seq) atlas of human blood immune cells. We found that IL1R2 is primarily expressed by monocytes and neutrophils in the blood of septic patients (Figure 1D–F). In addition, we found that IL1R1 expression is slightly upregulated on monocytes, but much lower than IL1R2 during sepsis (Figure S2A,B, Supporting Information). We also analyzed a publicly available mouse single‐cell RNA sequencing (scRNA‐seq) atlas of lungs and liver of sham and CLP mice. We found that IL1R2 is primarily expressed by monocyte‐derived macrophages and neutrophils in the lungs and liver of sham and CLP mice (Figure S2C–F, Supporting Information). While IL1R2 expression is very low in tissue resident macrophages, like alveolar macrophages (AMs) and Kupffer cells, in the lung and liver of sham and CLP mice (Figure S2C–F, Supporting Information). We further collected peripheral blood samples from sepsis patients and assessed IL1R2 expression on monocytes using multi‐color flow cytometry. Monocytes were classified into classical monocytes (CD11b⁺CD14⁺CD16^−^) and non‐classical monocytes (CD11b⁺CD14^−^CD16^−^). Flow cytometry analysis showed that IL1R2 expression in classical monocytes from sepsis patients was significantly higher than in healthy controls (Figure 1G). IL1R2 expression in non‐classical monocytes from sepsis patients showed an increased trend than in healthy controls (Figure 1H). We also validated that plasma sIL1R2 levels were significantly elevated in CLP‐induced septic mice compared to sham mice (Figure 1I). Consistent with previous studies, which demonstrated high surface expression and release of IL1R2 by monocytes and monocyte‐derived macrophages,^[^ ^17^ ^]^ these findings indicate that monocytes/macrophages are likely the primary source of sIL1R2 in the blood in septic patients and mice.

To determine whether sIL1R2 is released under inflammatory conditions, we examined its expression in macrophages undergoing pyroptosis. We found that intracellular IL1R2 levels significantly decreased in mouse peritoneal macrophages undergoing LPS and nigericin‐induced pyroptosis, while the levels of IL1R2 in the culture supernatants of pyroptotic macrophages increased markedly (Figure 1J). In contrast, sIL1R2 was undetectable in the culture supernatant of macrophages treated with LPS alone (Figure 1J). Confocal immunofluorescence confirmed that IL1R2 expression was significantly decreased in pyroptotic macrophages (Figure 1K). However, our results indicate that the GSDMD pore formation inhibitor, disulfiram, significantly reduces the cleavage of GSDMD with reduced N‐GSDMD expression. While disulfiram treatment did not influence the release of soluble IL1R2 in macrophages after being stimulated with LPS+ATP. This result suggests that sIL1R2 was released by other protein secretion pathways, rather than actively released from the GSDMD pore in the pyroptotic cell (Figure 1L). Collectively, these data suggest that sIL1R2 is linked to the severity of sepsis and is actively released from pyroptotic macrophages during inflammation.

### 2.2 Proteomic Screens Reveal IL1R2‐ENO1 Interaction

**Figure 2:**
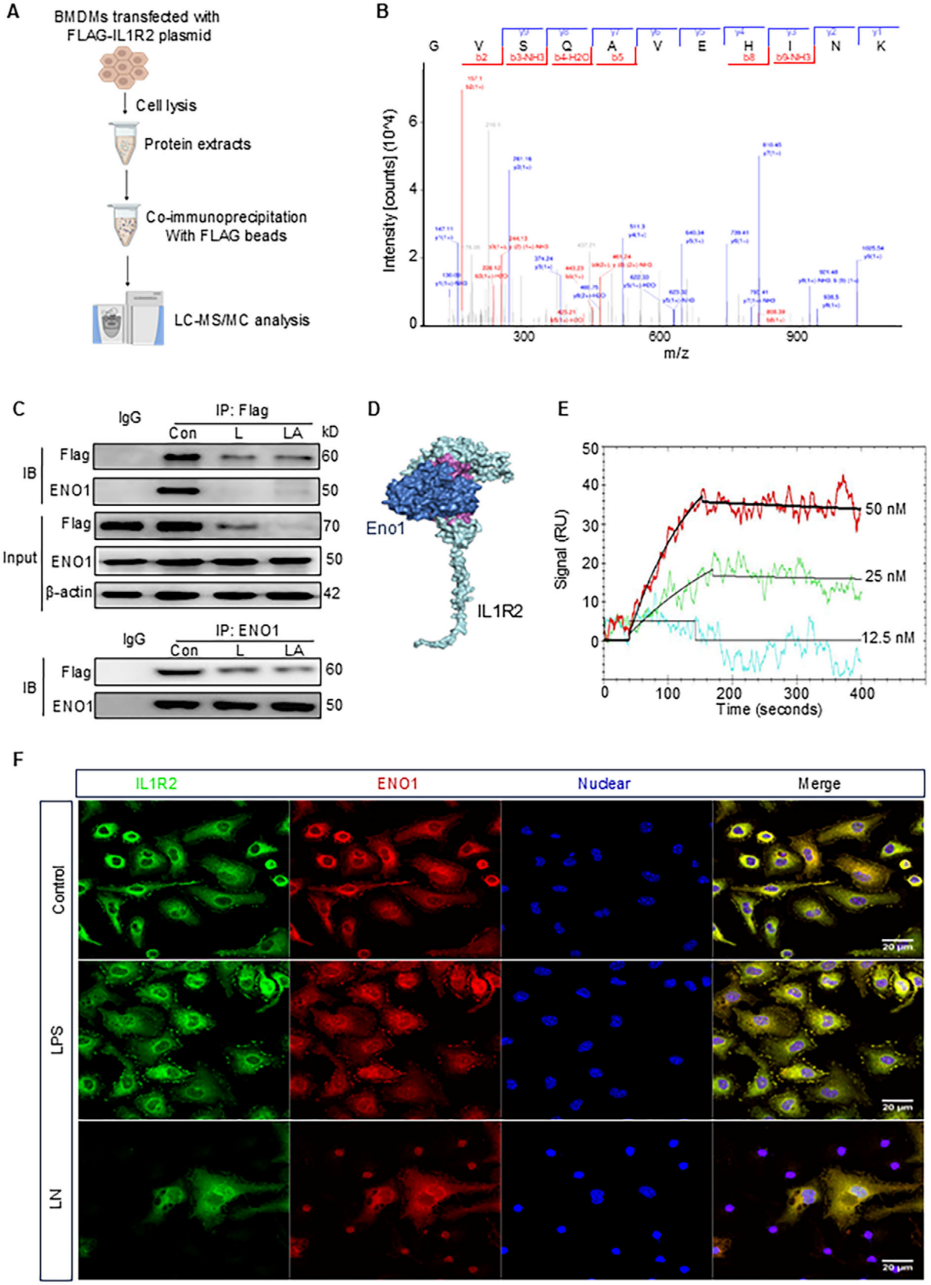
Proteomic screens elucidate IL1R2‐ENO1 interaction. A) Schematic workflow for identifying the direct binding partners of IL1R2 by affinity purification LC‐MS/MS. B) Mass spectrometry identification of ENO1 protein. C) Flag‐IL1R2 was immunoprecipitated in Flag‐IL1R2‐overexpressing iBMDMs, followed by immunoblotting with indicated antibodies (top). ENO1 was immunoprecipitated in Flag‐IL1R2‐overexpressing iBMDMs, followed by immunoblotting with indicated antibodies (bottom). Representative images from three independent experiments with similar results. D) Computational model of the interaction between mouse IL1R2 (blue) and ENO1 (light blue). E) Biacore analysis demonstrating binding of rmIL1R2 and rmENO1 with a *K_D_* of 1.99×10^−9^. F) PerC macrophages treated with or without LPS (1 µg mL^−1^) for 3 h, then followed with nigericin (5 µM) for 1 h. After stimulation, the macrophages were fixed and stained with anti‐IL1R2 Ab (red), anti‐ENO1 Ab (green), and DNA (Hoechst33342, blue), and the images were captured by confocal microscopy. Scale bar: 20 µm. Original magnification: 630×.

Building on our understanding of IL1R2 signaling and function,^[^ ^18^ ^]^ we hypothesized that IL1R2 may bind to proteins other than members of the IL1 family to modulate macrophage functions. To identify potential IL1R2‐binding partners in macrophages, we performed immunoprecipitation‐mass spectrometry (IP‐MS/MS) using iBMDMs overexpressing Flag‐tagged IL1R2 (**Figure** **2**A). IL1R2‐bound protein complexes were enriched from cell lysates by immunoprecipitation, subjected to stringent washes, and digested with trypsin for label‐free quantitative LC‐MS/MS analysis. Several proteins were enriched in the IL1R2‐bound fraction, including ENO1, ENO3, HSP90AB1, MIF, and others (Figure 2B; Table S3, Supporting Information). Notably, ENO1 had the highest protein score among all the identified proteins. We further validated the IL1R2‐ENO1 interaction via co‐IP assays in iBMDMs overexpressing Flag‐IL1R2 (Figure 2C). Interestingly, this interaction was absent in macrophages undergoing pyroptosis, as IL1R2 was released into the extracellular space (Figure 2C). Computational modeling supported the interaction between IL1R2 and ENO1, with the predicted complex exhibiting a surface interface area of 3147.6 Ȧ^2^ and a binding energy (∆iG) of −17.3 kcal mol^−1^ (Figure 2D; Table S4, Supporting Information). The negative value of ∆iG indicated a robust interaction of the complex. In addition, certain residues at the IL1R2–ENO1 interaction interface overlap with those at ENO1's substrate‐binding site, suggesting that IL‐1R2 may influence ENO1 enzymatic activity. The ENO1 residues at the interaction interface include D13, S14, R15, G16, N17, G38, A39, T41, I43, E48, R50, K54, M58, K60, S157, G160, D203, N206, V207, D209, S249, E250, F261, K262, S263, P264, and R400. However, IL1R2 does not directly bind to the active/catalytic site residues of ENO1 (E210 and K243), indicating that while the ENO1–IL1R2 interaction may not disrupt ENO1's catalytic activity, it could impair substrate binding. To confirm the direct interaction between IL1R2 and ENO1, we performed SPR assays using rmIL1R2 and rmENO1. Our result demonstrated that rmIL1R2 and rmENO1 showed a very strong interaction with a dissociation constant (K_D_) of 1.99 × 10^−9^ M (Figure 2E). Immunostaining in peritoneal macrophages further visualized the interaction between IL1R2 and ENO1. In macrophages treated with PBS or LPS, IL1R2 and ENO1 co‐localized at the plasma membrane and intracellular compartments (Figure 2F). However, this co‐localization was largely absent in pyroptotic macrophages (Figure 2F). Collectively, these data indicate that IL1R2 directly interacts with ENO1 in macrophages, potentially playing a role in macrophage function under non‐pyroptotic conditions.

### 2.3 IL1R2 Suppresses ENO1‐Mediated Glycolysis in Macrophages

**Figure 3:**
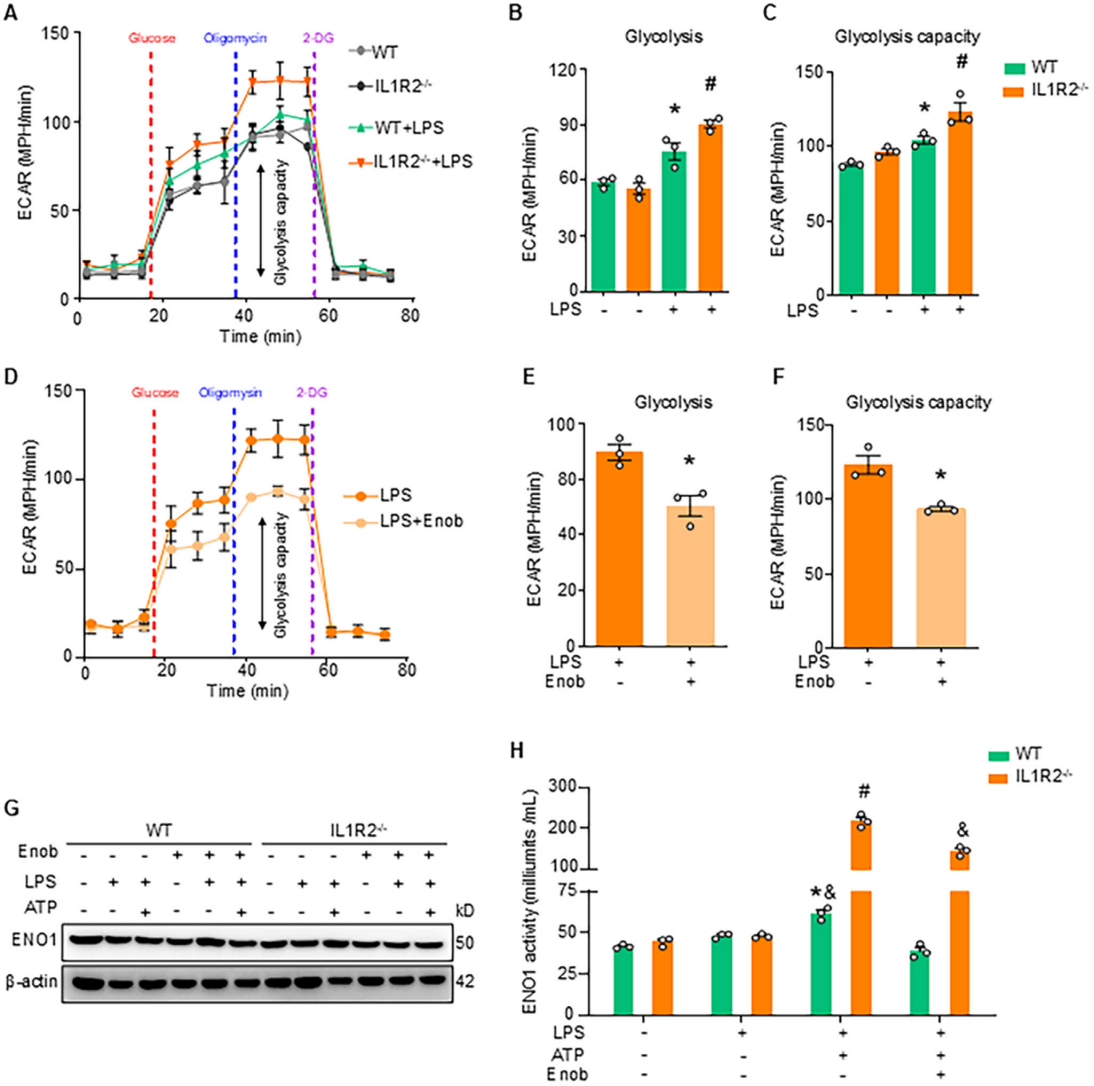
IL1R2 suppresses ENO1‐mediated glycolysis in macrophages after LPS stimulation. A–C) WT or IL1R2^−/‐^ primary PerC macrophages were stimulated without or with LPS (1 µg/mL) for 12 h. Extracellular acidification rate (ECAR) in macrophages as assessed by Seahorse assay. (A) Real‐time changes in the ECAR of macrophages after treatment with glucose, Oligomycin, and 2‐DG. Glycolysis capacity (double‐headed arrow) is shown in macrophages. (B) Glycolysis of macrophages was measured by real‐time changes in ECAR. (C) Glycolysis capacity of macrophages measured by real‐time changes in ECAR. Data are expressed as mean ± SEM and compared by one‐way ANOVA and Tukey's multiple comparisons test. *p < 0.05 versus WT+LPS (‐), ^#^ *p* < 0.05 versus WT+LPS (+). D–F) IL1R2^−/‐^ primary PerC macrophages were stimulated without or with LPS and treated with Enoblock (10 µM) for 12 h. ECAR in macrophages as assessed by Seahorse assay. (D) Real‐time changes in the ECAR of IL1R2^−/‐^ macrophages after treatment with glucose, Oligomycin, and 2‐DG. Glycolysis capacity (double‐headed arrow) is shown in macrophages. (E) Glycolysis of IL1R2^−/‐^ macrophages measured by real‐time changes in ECAR. (F) Glycolysis capacity of IL1R2^−/‐^ macrophages measured by real‐time changes in ECAR. Data are expressed as mean ± SEM and compared by Student's t test (unpaired). *p < 0.05 versus LPS (+), Eno (‐). G) WT and IL1R2^−/‐^ PerC macrophages treated with or without LPS (1 µg mL^−1^) for 3 h, then followed with ATP (5 mM) and Enoblock (10 µM) for 0.5 h. The expression of ENO1 in the whole cell lysis of these macrophages was measured by WB. Representative images from three independent experiments with similar results. H) The activity of ENO1 in these macrophages was measured by ENO1 activity kit. All experiments were repeated three times with similar results. Data are expressed as mean ± SEM and compared by two‐way ANOVA and Tukey's multiple comparisons test. *p < 0.05 versus WT LPS (‐) ATP (‐) Enob (‐), #*p* < 0.05 versus IL1R2^−/−^ LPS (‐) ATP (‐) Enob (‐), &*p* < 0.05 versus IL1R2^−/−^ LPS (+) ATP (+) Enob (‐).

ENO1 is an enzyme that catalyzes the conversion of 2‐phospho‐D‐glycerate into phosphoenolpyruvate during glycolysis and the reverse reaction in gluconeogenesis, processes critical for cellular function.^[^ ^16^ ^]^ The discovery of the interaction between IL1R2 and ENO1 helps us to hypothesize that IL1R2 modulates glycolysis via ENO1 regulation in macrophages. To test this, we used a Seahorse extracellular flux analyzer to monitor extracellular acidification rate (ECAR), a measure of glycolysis and glycolytic capacity in peritoneal macrophages isolated from WT and IL1R2^−/−^ mice. Under resting conditions, both WT and IL1R2^−/−^ macrophages showed similar baseline levels of glycolysis and glycolytic capacity (**Figure** **3**A–C). Upon LPS stimulation, glycolysis increased by 28.2% and 62.4% in WT and IL1R2^−/−^ macrophages, respectively, as indicated by the rise in ECAR. Additionally, glycolytic capacity increased by 18.4% and 27.7% in WT and IL1R2^−/−^ macrophages, respectively. Notably, IL1R2^−/−^ macrophages showed 19.3% higher glycolysis and 18.1% greater glycolytic capacity compared to WT macrophages following LPS stimulation (Figure 3A–C). These data suggest that IL1R2 plays a negative/inhibitory role in glycolysis of activated macrophages.

Given that IL1R2 interacts with ENO1, we investigated whether the inhibitory effect of IL1R2 on glycolysis is dependent on its interaction with ENO1. To test this, we used the ENO1 inhibitor, Enoblock, and performed the Seahorse experiments in IL1R2^−/−^ macrophages. Enoblock treatment decreased glycolysis by 27.5% and glycolytic capacity by 23.9% in LPS‐stimulated IL1R2^−/−^ macrophages compared to without Enoblock‐treated macrophages (Figure 3D–F). Next, we explored whether the inhibition of ENO1 by IL1R2 was mediated by the decreased expression or activity of ENO1. We assessed the gene expression of several key glycolytic enzymes in si‐IL1R2 iBMDMs following LPS stimulation and Enoblock treatment. Our results revealed that IL1R2 deficiency or Enoblock treatment did not significantly alter the gene expression levels of these enzymes, including ENO1 itself and PKM2, under LPS stimulation (Figure S3A, Supporting Information). Western blot analysis revealed that ENO1 protein expression was similar between WT and IL1R2^−/−^ macrophages treated with or without LPS or LPS+ATP (Figure 3G). Direct assessment of enolase activity in cell lysates showed that ENO1 activity did not increase in both WT and IL1R2^−/−^ macrophages after being stimulated with LPS for 3 h. However, ENO1 activity was increased in LPS+ATP‐stimulated WT macrophages and was notably higher in LPS+ATP‐stimulated IL1R2^−/−^ macrophages compared to WT (Figure 3H). Together, these data support the hypothesis that IL1R2 suppresses glycolysis in macrophages by inhibiting ENO1 activity, rather than by affecting its expression.

To evaluate the impact of IL1R2 on oxidative phosphorylation (OXPHOS), we assessed the mitochondrial OCR in WT and IL1R2^−/−^ peritoneal macrophages. Under resting conditions, both WT and IL1R2^−/−^ macrophages exhibited similar baseline levels of glucose oxidation and palmitic acid oxidation (Figure S3B,C, Supporting Information). After LPS stimulation, both WT and IL1R2^−/−^ macrophages showed a reduced oxidative state, as indicated by a decrease in basal or maximal respiratory capacity compared to PBS‐treated cells (Figure S3B–D, Supporting Information). However, no significant differences were observed in basal or maximal respiratory capacity between WT and IL1R2^−/−^ macrophages after LPS stimulation (Figure S3B–D, Supporting Information). These findings suggest that IL1R2 does not play a role in regulating oxidative phosphorylation in macrophages.

### 2.4 IL1R2 Suppresses GSDMD‐Mediated Pyroptosis by Targeting ENO1

**Figure 4:**
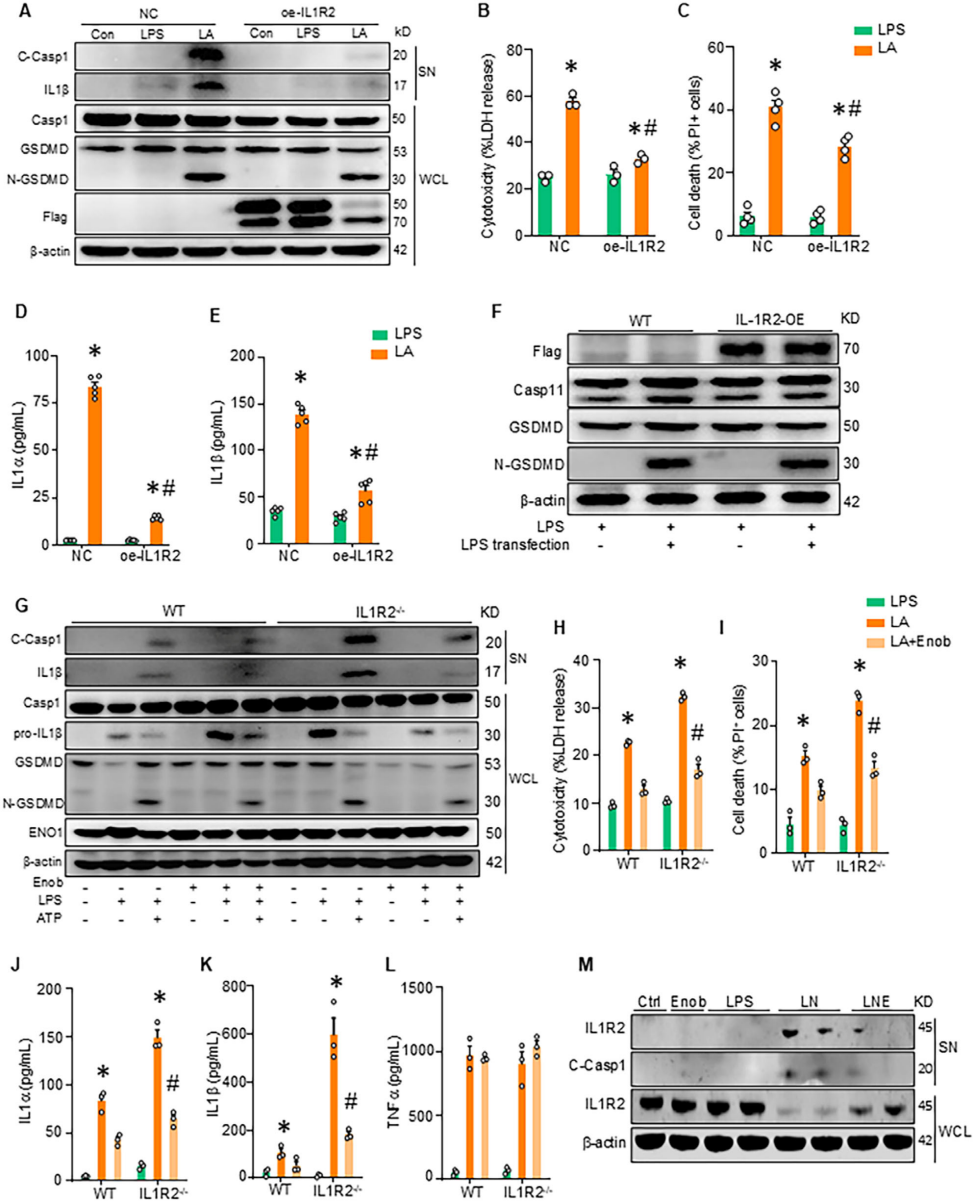
IL1R2 suppresses GSDMD‐mediated pyroptosis in macrophages through ENO1. A–E) iBMDMs were transfected with a flag‐IL1R2 overexpression plasmid (oe‐IL1R2) or a negative control (NC). After transfection, the cells were treated with LPS (1 µg/mL) for 3 h, then followed by ATP (5 mM) for 0.5 h. (A) The expressions of cleaved‐Caspase1(C‐Casp1) and IL1β in the supernatants (SN), and the expression of casp1, GSDMD, N‐GSDMD, and IL1R2 (Flag) in the whole cell lysis (WCL) of these iBMDMs were measured. Representative images from three independent experiments with similar results. (B) The levels of LDH in the culture supernatants of iBMDMs were measured. (C) The percentage of cell death (PI^+^ cells) was checked in these iBMDMs. (D, E) The levels of IL1α and IL1β in the culture supernatants of iBMDMs were measured by ELISA kits. Data are expressed as mean ± SEM and compared by two‐way ANOVA and Tukey's multiple comparisons test. *p < 0.05 versus NC+LPS, ^#^p < 0.05 versus NC+LA. F) iBMDMs were transfected with flag‐IL1R2 overexpression plasmid (oe‐IL1R2) or negative control (NC). After transfection, the cells were treated with LPS (1 µg/mL) for 6 h, followed by LPS (5 µg mL^−1^) for 6 h. The expressions of Caspase11(Casp11), GSDMD, N‐GSDMD, and IL1R2 (Flag) in the whole cell lysis of these iBMDMs were measured. Representative images from three independent experiments with similar results. G–L) WT and IL1R2^−/−^ PerC macrophages treated with LPS (1 µg/mL) for 3 h, then followed with ATP (5 mM) and Enoblock (10 µM) for 0.5 h. (G) The expression of cleaved‐caspase1(C‐Casp1) and IL1β in the supernatants (SN), and the expression of casp1, pro‐IL1β, GSDMD, N‐GSDMD, and ENO1 in the WCL of these macrophages were measured. Representative images from three independent experiments with similar results. (H) The levels of LDH in the culture supernatants of macrophages were measured by kits. (I) The percentage of cell death (PI^+^ cells) was checked in these macrophages. (J‐L) The levels of IL1α (J), IL1β (K), and TNFα (L) in the culture supernatants of macrophages were measured by ELISA kits. Data are expressed as mean ± SEM and compared by two‐way ANOVA and Tukey's multiple comparisons test. *p < 0.05 versus WT+LA, ^#^ *p* < 0.05 versus IL1R2^−/−^+LA. LA, LPS with ATP. M) WT PerC macrophages treated with LPS (1 µg mL^−1^) for 3 h, then followed with nigericin (5 µM) and Enoblock (10 µM) for 0.5 h. The expressions of cleaved‐caspase1(C‐Casp1) and IL1R2 in the supernatants, and the expression of IL1R2 and β‐actin in the WCL of these macrophages were measured. Representative images from three independent experiments with similar results.

Recent studies show that glycolytic enzymes regulate inflammasome activation.^[^ ^19^ ^]^ To explore the role of metabolic regulation by IL1R2‐ENO1 in inflammatory responses, we employed a two‐signal model for caspase‐1‐mediated canonical pyroptosis, in which macrophages were primed with LPS (signal 1) and subsequently stimulated with ATP (signal 2). First, we used siRNA transfection to knock down IL1R2 expression in iBMDMs. Knockdown of IL1R2 (si‐IL1R2 iBMDMs) resulted in increased caspase1 and GSDMD cleavage (Figure S4A, Supporting Information), along with heightened cell death, as evidenced by higher LDH release and an elevated percentage of PI^+^ cells, compared to control (si‐NC) iBMDMs (Figure S4B,C, Supporting Information). Furthermore, the production of IL1α and IL1β was significantly elevated in si‐IL1R2 BMDMs compared to si‐NC iBMDMs (Figure S4D,E, Supporting Information). We then overexpressed IL1R2 in BMDMs by plasmid transfection. Overexpression of IL1R2 (oe‐IL1R2 iBMDMs) led to reduced caspase1 and GSDMD cleavage (**Figure** **4**A), along with decreased cell death, as indicated by lower LDH release and a reduced percentage of PI^+^ cells, compared to the control group (oe‐NC iBMDMs) (Figure 4B,C). Additionally, IL1α and IL1β production were significantly reduced in oe‐IL1R2 iBMDMs compared to oe‐NC iBMDMs (Figure 4D,E). Next, we further investigated whether IL1R2 regulates caspase‐11‐mediated non‐canonical pyroptosis in macrophages by transfecting oe‐IL1R2 iBMDMs with high doses of LPS. Overexpression of IL1R2 did not affect caspase‐11 expression and GSDMD cleavage (Figure 4F). Therefore, these data indicate that IL1R2 inhibits caspase‐1‐mediated canonical pyroptosis in macrophages.

We also investigated the role of ENO1 on GSDMD‐mediated pyroptosis in macrophages. First, using siRNA transfection, we generated ENO1 knockdown iBMDMs and induced pyroptosis by stimulating with LPS+ATP. Knockdown of ENO1 (si‐ENO1 iBMDMs) resulted in increased caspase1 and GSDMD cleavage (Figure S4F, Supporting Information), as well as enhanced cell death, as indicated by higher LDH release and an elevated percentage of PI^+^ cells compared to control (si‐NC) iBMDMs (Figure S4G,H, Supporting Information). The production of IL1α and IL1β was significantly increased in si‐ENO1 iBMDMs compared to si‐NC (Figure S4I,J, Supporting Information). These data indicate that ENO1 promotes GSDMD‐mediated pyroptosis in macrophages. Next, we investigated whether the inhibition of pyroptosis by IL1R2 was mediated through ENO1. We induced pyroptosis by stimulating IL1R2^−/−^ primary peritoneal macrophages with LPS and ATP, while using Enoblock to inhibit ENO1. IL1R2^−/−^ macrophages exhibited enhanced caspase 1 and GSDMD cleavage (Figure 4G), and increased cell death compared to WT macrophages (Figure 4H,I). IL1α and IL1β release was also significantly elevated in IL1R2^−/−^ macrophages compared to WT macrophages (Figure 4J,K). Notably, treatment of IL1R2^−/−^ cells with Enoblock resulted in decreased caspase 1 and GSDMD cleavage, cell death, and IL1α and IL1β release (Figure 4G–K). Interestingly, TNFα levels were similarly increased in both WT and IL1R2^−/−^ pyroptotic macrophages and were not inhibited by Enoblock (Figure 4L). These data suggest that IL1R2 inhibits caspase1‐dependent inflammasome activation via ENO1.

Following inflammasome activation, caspase‐1 specifically cleaves IL1R2 to promote IL1α‐dependent responses.^[^ ^20^ ^]^ Given our observation of decreased IL1R2 expression and increased caspase1 activation in pyroptotic macrophages, we hypothesized that inhibiting ENO1 could reduce caspase1‐mediated IL1R2 cleavage and release in pyroptotic macrophages. Interestingly, our results demonstrated that IL1R2 expression was increased in Enoblock‐treated pyroptotic macrophages (Figure 4M). In contrast, caspase1 activation and IL1R2 release were significantly decreased in Enoblock‐treated pyroptotic macrophages (Figure 4M). Thus, our findings suggest that ENO1 plays a role in the regulation of IL1R2 expression and caspase‐1 activity in pyroptotic macrophages.

### 2.5 IL1R2 Deficiency Exacerbates Organ Damage in Sepsis

**Figure 5:**
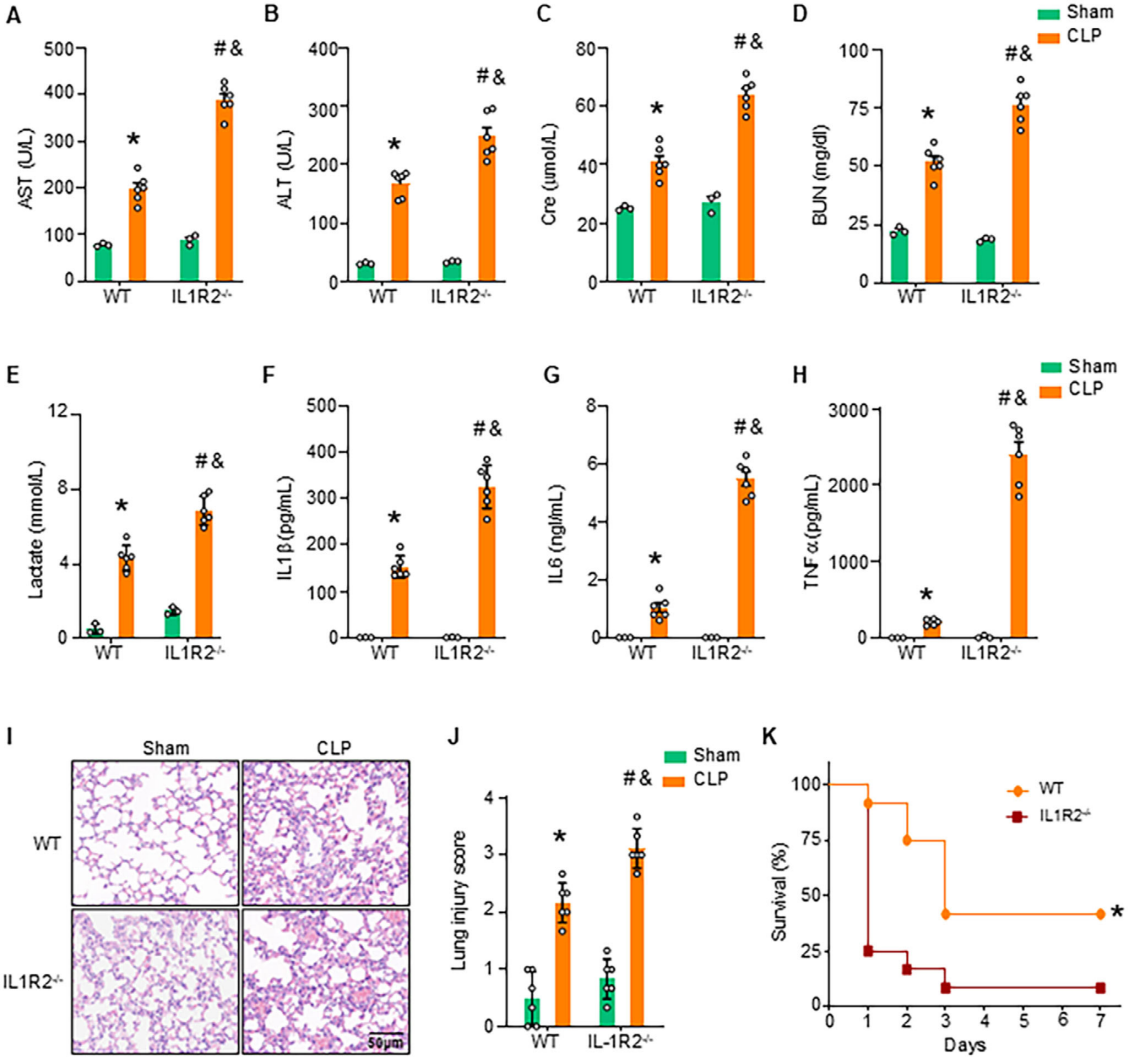
IL1R2 deficiency exacerbates organ damage in septic mice. A–J) WT and IL1R2^−/‐^ mice were assigned to sham or CLP‐induced sepsis. After 20 h, blood and lung tissue were collected. (A‐D) The plasma levels of AST (A), ALT (B), Cre (C), and BUN (D) were measured by commercial kits. (E) The plasma levels of lactate were measured using by commercial kit. (F‐H) The plasma levels of IL1β (F), IL6 (G), and TNFα (H) were measured by ELISA kits. Experiments were performed three times, and all data were used for analysis. (I, J) Lungs were embedded in paraffin, sectioned, and analyzed for histologic injury using H&E staining and analyzed using lung injury scoring. Data are expressed as mean ± SEM and compared by two‐way ANOVA and Tukey's multiple comparisons test. *p < 0.05 versus sham WT, ^#^ *p* < 0.05 versus sham IL1R2^−/−^, ^&^ *p* < 0.05 versus CLP WT. K) WT or IL1R2^−/‐^ mice subjected to CLP‐induced sepsis and monitored for 7 days for humane endpoints, and differences in survival were determined using Kaplan‐Meier survival plots and a log‐rank test. *n* = 12 mice/group. Hazard ratios (HR): WT, 0.36; IL1R2^−/−^, 2.75. *p < 0.05 versus WT.

To determine the role of IL1R2 in sepsis, we examined its effects in WT and IL1R2^−/−^ mice subjected to CLP‐induced sepsis. IL1R2^−/−^ septic mice demonstrated aggravated liver and kidney injury, indicated by elevated plasma levels of AST, ALT, Cre, and BUN compared to WT septic mice (**Figure** **5**A–D). Additionally, IL1R2^−/−^ septic mice exhibited significantly higher plasma lactate levels compared to WT septic mice (Figure 5E). Furthermore, IL1R2^−/−^ septic mice showed exacerbated systemic inflammation, as evidenced by increased plasma concentrations of pro‐inflammatory cytokines IL1β, IL6, and TNFα compared to WT septic mice (Figure 5F–H). Next, lung sections stained with HE, harvested 20 h after CLP, revealed significantly greater lung tissue destruction and inflammation in IL1R2^−/−^ mice compared to WT mice (Figure 5I,J). Finally, IL1R2^−/−^ mice showed a higher mortality rate of 91.7% compared to 58.3% in WT mice following CLP (Figure 5K). Collectively, these findings indicate that IL1R2 deficiency aggravates organ injury and inflammation in CLP‐induced sepsis.

To further investigate the role of IL1R2 in protecting against sepsis, we used a macrophage‐specific IL1R2 conditional knockdown (IL1R2^CKD^) mouse model of CLP‐induced sepsis. IL1R2^CKD^ septic mice showed aggravated liver and kidney injury, with higher plasma levels of AST, ALT, Cre, and BUN compared to WT septic mice (Figure S6A–D, Supporting Information). Similarly, plasma lactate levels were significantly elevated in IL1R2^CKD^ septic mice (Figure S6E, Supporting Information). These mice also exhibited increased plasma levels of IL1β, IL6, and TNFα, indicating heightened systemic inflammation (Figure S6F–H, Supporting Information). Histopathological analysis of lung sections stained with HE, harvested 20 h after CLP, revealed significantly greater lung tissue destruction and inflammation in IL1R2^CKD^ septic mice compared to WT mice (Figure S6I,J, Supporting Information). Together, these data suggest that macrophage‐specific knockdown of IL1R2 exacerbates organ damage and inflammation in CLP‐induced septic mice.

### 2.6 Inhibiting ENO1 Improves Sepsis

**Figure 6:**
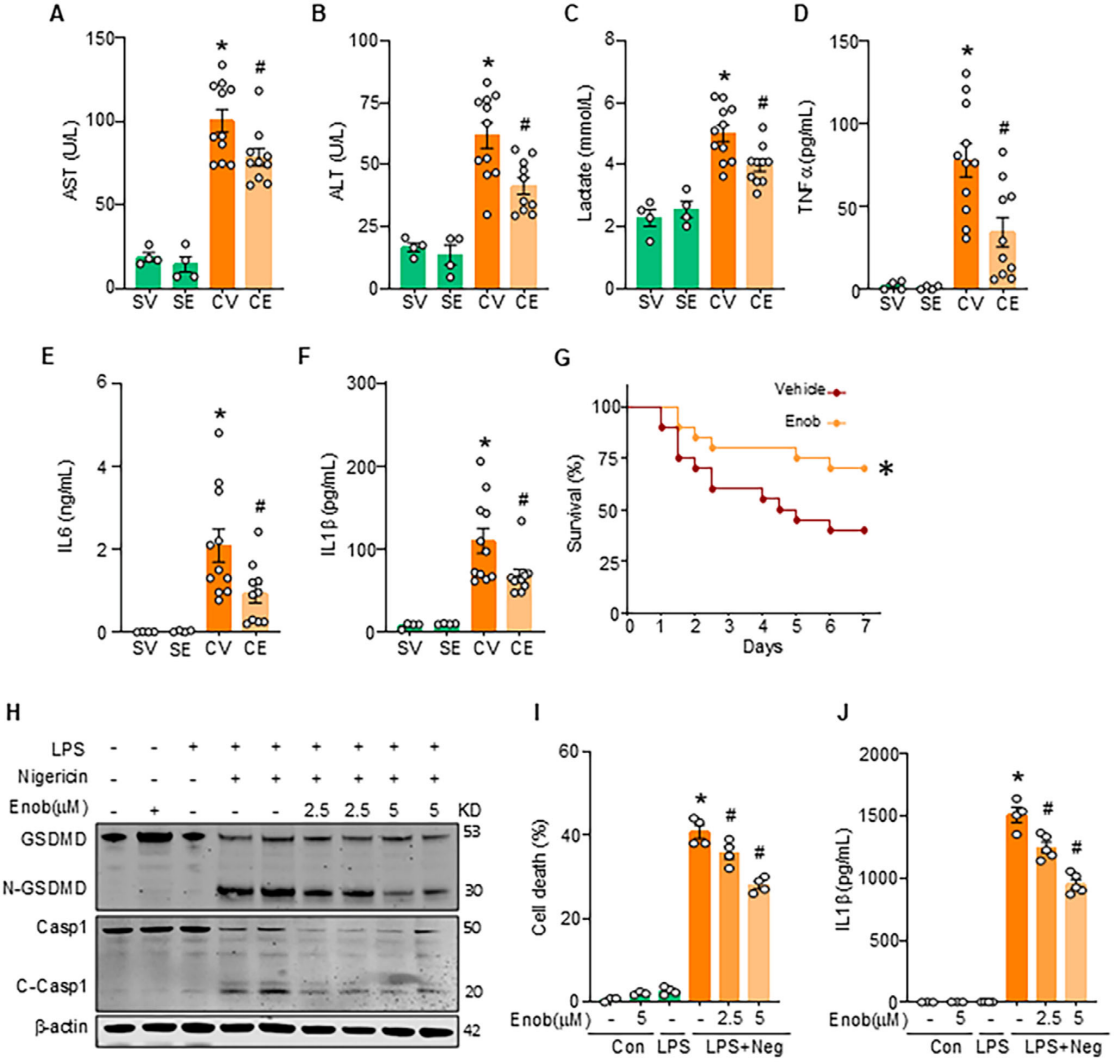
ENO1 inhibition improves sepsis in CLP‐induced septic mice. A–F) WT mice were subjected to CLP‐induced sepsis with Enoblock treatment (5 mg kg^−1^) or vehicle (volume‐equivalent). After 20 h, blood and lung tissue were collected for respective analyses. (A, B) The plasma levels of AST (A) and ALT (B) were measured by commercial kits. (C) The plasma levels of lactate were measured using by commercial kit. (D‐F) The plasma levels of TNFα (D), IL6 (E), and IL1β (F) were measured by ELISA kits. Experiments were performed 3 times, and all data were used for analysis. Data are expressed as mean ± SEM and compared by one‐way ANOVA and Tukey's multiple comparisons test. *p < 0.05 versus SV, ^#^ *p* < 0.05 versus CV. SV, Sham+vehicle; CV, CLP+vehicle; SE, Sham+Enoblock; CE, CLP+ Enoblock. G) WT mice subjected to CLP‐induced sepsis with Enoblock treatment (5 mg/kg) or vehicle (volume equivalent) and monitored for 7 days for humane endpoints, and differences in survival were determined using Kaplan‐Meier survival plots and a log‐rank test. *n* = 20 mice/group. Hazard ratios: CL+vehicle, 2.51; CLP+ Enoblock, 0.40. *p < 0.05 versus CLP vehicle. H–J) WT PerC macrophages were treated with LPS (1 µg/mL) for 3 h, then followed with nigericin (5 µM) and indicated dose of Enoblock for 1 h. (H) The expression of cleaved‐caspase1(C‐Casp1), caspase1, GSDMD, and N‐GSDMD, in the whole cell lysis of these macrophages, was measured by WB. Representative images from three independent experiments with similar results. (I) The levels of LDH in the culture supernatants of macrophages were measured by kits. (J) The levels of IL1β in the culture supernatants of macrophages were measured by ELISA kits. Data are expressed as mean ± SEM and compared by one‐way ANOVA and Tukey's multiple comparisons test. * *p* < 0.05 versus LPS+ nigericin (‐) Enob (‐), ^#^ *p* < 0.05 versus LPS+ nigericin (+) Enob (+). Enob, Enobock.

To better translate the significance of the IL1R2‐ENO1 interaction in attenuating pyroptosis‐induced inflammation in vivo, we examined the effects of the ENO1 inhibitor, Enoblock, in a CLP‐induced sepsis. We found that Enoblock treatment mitigated liver injury, as evidenced by significantly lower plasma levels of AST and ALT compared to vehicle‐treated septic mice (**Figure** **6**A,B). Additionally, plasma lactate levels were lower in Enoblock‐treated septic mice compared to the vehicle group (Figure 6C). Moreover, Enoblock treatment reduced systemic inflammation, as shown by decreased plasma levels of the pro‐inflammatory cytokines IL1β, IL6, and TNFα in comparison to vehicle‐treated WT septic mice (Figure 6D–F). Next, we found that Enoblock treatment improved the survival rate from 40% in vehicle‐treated WT mice to 70% in Enoblock‐treated mice following CLP (Figure 6G). Finally, we investigated pyroptosis in vitro by stimulating WT primary peritoneal macrophages with LPS and nigericin and subsequently treating them with Enoblock to inhibit ENO1. Enoblock‐treated macrophages displayed reduced caspase1 cleavage, GSDMD cleavage, and cell death compared to untreated WT macrophages (Figure 6H,I). IL1β production was significantly decreased in Enoblock‐treated macrophages compared to controls (Figure 6J). These findings collectively support that ENO1 inhibition effectively reduces systemic inflammation, protects against organ injury, and improves survival in sepsis. Furthermore, Enoblock treatment improved the survival rate from 27% in IL1R2^CKD^ mice to 64% in Enoblock‐treated mice following CLP (Figure S6H, Supporting Information). This finding supports the conclusion that IL1R2 protects mice from sepsis by inhibiting ENO1.

## 3 Discussion

**Figure 7:**
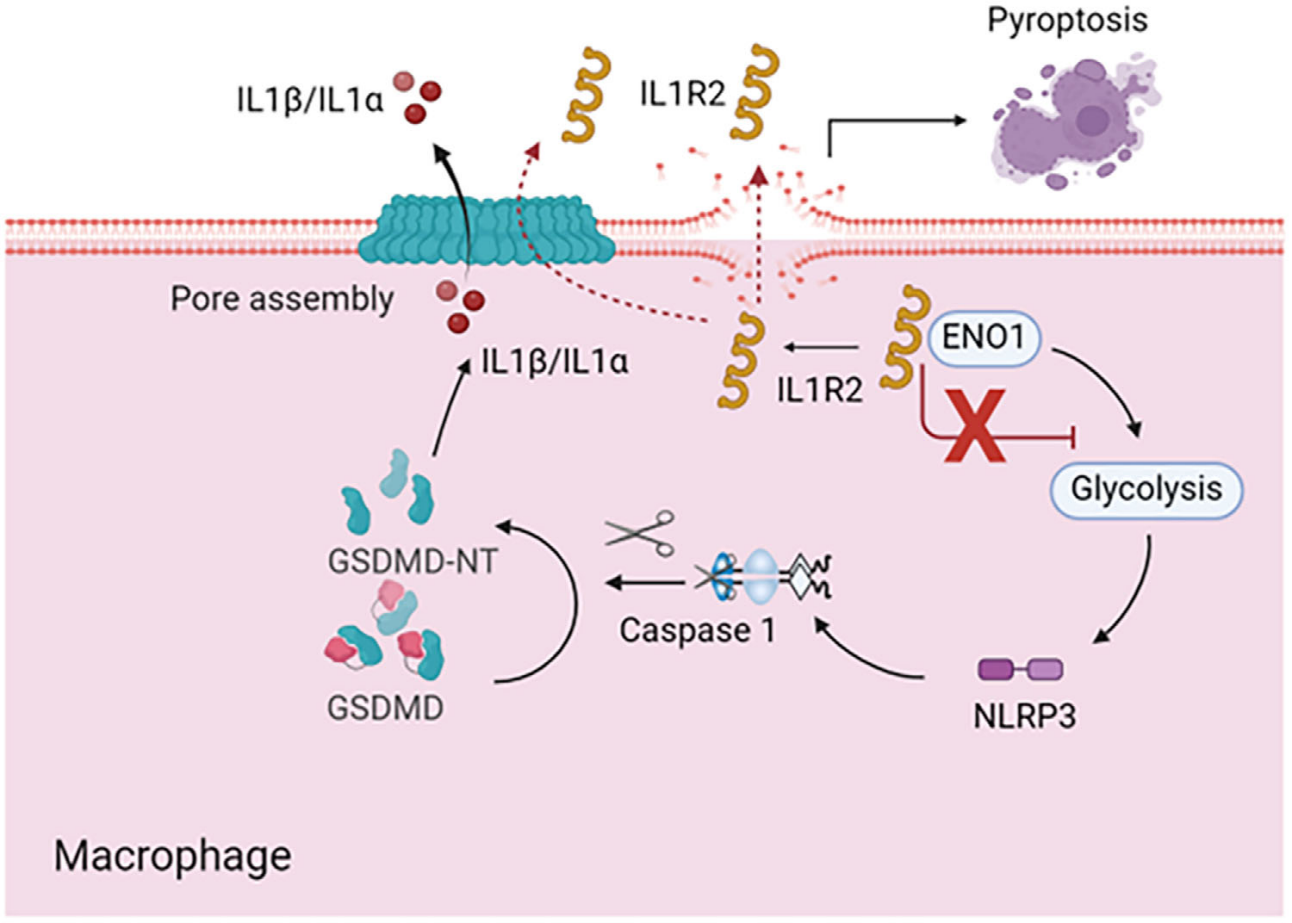
Summary of findings. We discovered that intracellular IL1R2 negatively regulates inflammatory cell death and inflammation by inhibiting glycolysis in sepsis. Soluble IL1R2 is released from macrophages undergoing pyroptosis. IL1R2 acts as a novel negative regulator of glycolysis by interacting with ENO1, thereby inhibiting GSDMD‐mediated pyroptosis and inflammation. The pharmacologic and genetic inhibition of ENO1 suppressed GSDMD‐mediated pyroptosis by downregulating glycolysis‐mediated caspase‐1 activation. These results suggest that IL1R2‐ENO1 interaction holds potential as a therapeutic intervention for sepsis.

As a decoy receptor, IL1R2 plays a pivotal role in various pathological conditions, including infections, tumors, inflammatory and autoimmune diseases, primarily by inhibiting the IL1 signaling pathway.^[^ ^21^ ^]^ However, current knowledge of IL1R2's role in inflammation regulation has been limited to its blockade of IL1 signaling. In the present study, we have elucidated a novel mechanism by which IL1R2 modulates inflammation by inhibiting glycolysis during sepsis. (**Figure** **7**) We first observed elevated levels of sIL1R2 in the plasma of both septic patients and mice, suggesting its release into the extracellular space in sepsis. Further experiments identified IL1R2 as a novel regulator of glycolysis through its interaction with ENO1, thereby inhibiting caspase1‐mediated canonical pyroptosis and inflammation and protecting mice from sepsis‐induced organ injury and death. Importantly, both pharmacologic and genetic inhibition of ENO1 suppressed GSDMD‐mediated pyroptosis by downregulating glycolysis‐driven caspase1 activation. Our findings indicate that IL1R2‐ENO1 interaction holds promise as a therapeutic intervention for sepsis and may modulate the inflammatory response associated with infection.

The IL1R2 receptor was first identified in 1991 as a non‐signal‐transducing decoy for IL1.^[^ ^10^ ^]^ Despite its ability to independently bind both IL1α and IL1β, IL1R2 is unable to initiate downstream signaling pathways due to the absence of a TIR domain.^[^ ^10^, ^21^ ^]^ Consequently, IL1R2 competitively inhibits IL1‐mediated signaling, thus attenuating inflammatory signals in immune cells.^[^ ^21b^ ^]^ IL1R2 exists two distinct forms: membrane‐bound IL1R2(mIL1R2) and soluble IL1R2(sIL1R2).^[^ ^21b^ ^]^ sIL1R2 effectively binds IL1, comparable to the membrane receptor, and serves as a negative regulator of IL1 activity by counteracting the systemic effects of IL1 released from inflammatory sites.^[^ ^22^ ^]^ sIL1R2 has been detected in cell culture supernatants and in biological fluids under various pathological conditions, such as sepsis.^[^ ^23^ ^]^ Previous studies demonstrated that sIL1R2 is elevated in the plasma of septic patients, and sIL1R2 can be utilized as a diagnostic marker for sepsis and can even differentiate between gram‐negative and gram‐positive bacterial infections.^[^ ^23^, ^24^ ^]^ In our present study, we further discovered that the plasma level of sIL1R2 serves as a biomarker of sepsis, correlating with the severity and outcome of sepsis. Additionally, sIL1R2 has been shown to be correlated with other inflammatory conditions, like autoimmune diseases and cancer.^[^ ^21^, ^23^ ^]^ However, the primary source of sIL1R2 remains unclear, as it can originate from multiple mechanisms based on current studies. sIL1R2 is typically generated through the cleavage of mIL1R2's extracellular domain by metalloproteinase (shredded IL1R2),^[^ ^21^, ^25^ ^]^ alternative splicing of sIL1R2 mRNA to yield a soluble isoform, and direct release from cells under inflammatory or pathological conditions (cytoplasmic IL1R2).^[^ ^17^, ^23^, ^25^, ^26^ ^]^ In our study, we demonstrated that sIL1R2 is released from macrophages undergoing caspase 1‐mediated pyroptosis. However, GSDMD pore formation inhibitor, disulfiram, treatment did not influence the release of soluble IL1R2 in pyroptotic macrophages, suggesting that sIL1R2 was released by other protein secretion pathways, rather than being released from the GSDMD pore in pyroptotic cells. Additional research is needed to clarify the specific forms of sIL1R2 that are released, the precise mechanisms by which it is generated and transported, and the regulation pathways of sIL1R2 release.

As is well known, IL1 receptors are key regulators of the IL1 signaling pathway during the immune response. The IL1R complex is a multi‐component structure that includes two types of IL1‐binding receptors—IL1R1 and IL1R2—as well as an accessory receptor, IL1RAcP. Under physiological conditions, IL1R1 is expressed in non‐immune cells such as endothelial cells,^[^ ^27^ ^]^ whereas IL1R2 is mainly present in immune cells, like macrophages or monocytes.^[^ ^28^ ^]^ During infection, IL1α and IL1β bind to IL1R1 and activate downstream signaling cascades, including the NF‐κB and MAPK pathways, thereby amplifying the inflammatory response. Soluble IL1R2 is released from macrophages and acts as a decoy receptor, blocking IL1α/β–IL1R1 signaling to control IL1‐mediated systemic hyperinflammation in non‐immune cells. Lin et al utilized IL1R2 knockout mice to demonstrate that IL1R2 in cardiomyocytes protects cardiac organoids from apoptosis by reducing IL17RA expression.^[^ ^29^ ^]^ Along with our current study strongly supports that IL1R2 plays a broad protective role in mitigating sepsis‐induced injury across multiple organs. Despite both membrane‐bound and soluble IL1R2 playing important roles in suppressing IL1‐mediated signaling in various inflammatory disorders, IL1R2‐based therapeutics have not yet been reported.^[^ ^3^, ^8^, ^21^ ^]^ More recently, studies employing IL1R2‐neutralizing antibodies have revealed that IL1R2 orchestrates an immune‐suppressive tumor microenvironment by upregulating PD‐L1 expression, thereby enhancing the efficacy of anti‐PD‐1 therapy in triple‐negative breast cancer.^[^ ^30^ ^]^ Although elevated plasma levels of sIL1R2 are observed in septic patients, suggesting a role in neutralizing extracellular IL1 activity, clinical trials targeting IL1 have shown limited efficacy in sepsis.^[^ ^3^, ^6^ ^]^ This raises questions about the relative contributions of extracellular versus intracellular IL1R2 in modulating IL1‐mediated inflammation. Previous studies indicate that cytoplasmic IL1R2 binds to pro‐IL1α to inhibit its activity under normal conditions, but during inflammatory necrosis or caspase‐1 activation, IL1R2 is degraded, releasing pro‐IL1α.^[^ ^20^ ^]^ Whether cytoplasmic IL1R2 has additional roles in sepsis beyond this known function remains unclear. In our study, we have uncovered a novel regulatory function where IL1R2 influences the duration of inflammation by modulating cellular metabolism. Specifically, we observed that the absence of IL1R2 led to enhanced glycolysis in macrophages undergoing pyroptosis, resulting in the cleavage of caspase‐1 and GSDMD that promote IL1 production and inflammation. Importantly, through IP‐MS/MS screen, we discovered that ENO1, a key enzyme in the glycolytic pathway, is a novel interaction partner of IL1R2. Additionally, we demonstrated that IL1R2 directly binds to ENO1, inhibiting its enzymatic activity and thereby influencing glycolysis in macrophages under inflammatory conditions. Furthermore, pharmacologic inhibition or genetic deletion of ENO1 restricts GSDMD‐mediated cell pyroptosis and IL1 release in a similar manner to IL1R2 overexpression. While IL1R2 deficiency or ENO1 inhibition did not significantly alter the glycolysis‐related gene expression, including ENO1 itself and PKM2, under LPS stimulation. Together, these findings emphasize that the IL1R2–ENO1 interaction represents a specific mechanism by which IL1R2 modulates glycolysis and subsequently impacts pyroptosis. Therefore, our study expands the understanding of IL1R2 beyond its traditional role as a decoy receptor and highlights its involvement in regulating cellular metabolism and inflammatory responses. Future research exploring the precise mechanisms underlying IL1R2‐ENO1 interaction, such as structural studies or mechanistic assays, could provide deeper insights into how this interaction modulates immune function and inflammation.

In recent years, it has become clear that cellular metabolism affects immune cell function and modulates inflammation in sepsis.^[^ ^12b^ ^]^ During the hyperinflammatory phase of sepsis, there is a notable shift in energy metabolism where glycolysis predominates over oxidative phosphorylation, despite adequate oxygen availability.^[^ ^12a^ ^]^ Previous studies demonstrated that glycolysis serves a beneficial role by augmenting the production of metabolic intermediates essential for cellular biosynthesis and energy requirements. This metabolic shift supports processes such as cell growth, proliferation, and differentiation. However, mitochondrial dysfunction that develops in later stages prevents the restoration of oxidative phosphorylation and disrupts metabolic homeostasis, contributing to organ dysfunction in sepsis.^[^ ^12b^ ^]^ Previous studies showed that intervention of glycolysis can reduce inflammation and improve organ injury and survival in septic mice, suggesting that regulation of glycolysis is important for the regulation of inflammation and maintenance of immune homeostasis and health.^[^ ^13^, ^14^, ^15^ ^]^ Besides demonstrating that IL1R2 regulates glycolysis‐mediated inflammation by interacting with ENO1, our study highlights that targeting ENO1 can beneficially curb inflammation during sepsis. We first demonstrate that genetic reduction of ENO1 reduces GSDMD‐mediated pyroptosis and IL1 release in macrophages. Inhibition of ENO1with the inhibitor, Enoblock, reduces inflammation, organ injury, and improves survival in septic mice. Previous studies have shown that ENO1 inhibition ameliorates experimental hypoxic pulmonary hypertension by improving endothelial and mitochondrial function via PI3K‐Akt‐mTOR pathway.^[^ ^31^ ^]^ Other studies have highlighted ENO1's role in tissue remodeling during *Mycobacterium tuberculosis* infection, doxorubicin‐induced cardiomyocyte apoptosis, and cardiac remodeling.^[^ ^16^ ^]^ These findings strongly suggest that targeting ENO1 can mitigate inflammation in sepsis and other inflammatory diseases, offering potential for novel treatments focused on “metabolite correction.” Furthermore, specific peptides or small‐molecule inhibitors that target the interaction site of IL1R2‐ENO1 are currently being explored with the objective of achieving a more effective treatment of sepsis. Substantial studies have demonstrated that pyroptosis, IL1 signaling, and metabolic reprogramming are key features of various inflammatory conditions, including sterile inflammation (such as cancer and ischemia‐reperfusion injury) and autoimmune diseases. Existing literature also shows that IL1R2 and ENO1 play essential roles in multiple diseases.^[^ ^16^, ^21^ ^]^ We speculate that targeting the IL1R2–ENO1 regulatory axis may offer a novel therapeutic approach for a broad range of inflammatory conditions.

One limitation of our study is its primary focus on macrophages, despite IL1R2 also being expressed on neutrophils and other immune cells. The contributions of these cells to IL1R2‐mediated effects remain unclear. Investigating IL1R2 function in neutrophils and other immune cells, particularly its role in immune regulation and inflammatory resolution, would provide a more comprehensive understanding of its therapeutic potential. Additionally, our use of young male mice may not fully recapitulate the complexity of sepsis in older adults who are more susceptible to severe outcomes. Recognizing that sex differences may influence immune responses and disease progression, future studies will include both male and female mice to investigate potential sex‐based differences and assess whether targeting the IL1R2‐ENO1 pathway can offer protection against sepsis in aging mice. Given that Sepsis is a complex syndrome with diverse etiologies and patient demographics. Our study primarily addressed a specific experimental model of sepsis induced by CLP. Future research should encompass various sepsis models and patient populations to assess the generalizability of our findings.

In summary, our study uncovers a novel mechanism by which IL1R2 regulates inflammation by inhibiting glycolysis in sepsis. We first discovered that sIL1R2 is released from macrophages undergoing pyroptosis and identified IL1R2 as a novel regulator of glycolysis through its interaction with ENO1. This interaction inhibits GSDMD‐mediated pyroptosis and inflammation. Importantly, the pharmacologic and genetic inhibition of ENO1 suppressed GSDMD‐mediated pyroptosis by downregulating glycolysis‐driven caspase‐1 activation. Our results suggest that IL1R2‐ENO1 interaction holds potential as a new therapeutic intervention for sepsis and helps modulate inflammatory responses.

## 4 Experimental Section

### Human Materials

Patients were diagnosed with sepsis according to the sepsis‐3 definitions.^[^ ^1^ ^]^ Exclusion criteria were age <18 years, preexisting immunosuppression, being a transplant recipient, or having carcinoma. A total of 123 patients (31 with sepsis without shock and 92 with septic shock) from the intensive care unit (ICU) at the Third Xiangya Hospital of Central South University, between March 2018 and October 2019, met these criteria. Additionally, 29 patients admitted to the ICU for non‐infectious conditions were recruited as controls. The primary endpoint was the diagnosis of septic shock within the first 1–2 days of ICU stay, and the secondary endpoint was 28‐day all‐cause mortality. Plasma samples were obtained on day 1 when these patients were newly diagnosed with sepsis upon ICU admission. Blood was drawn into citrate (0.129 M) anticoagulants, followed by plasma collection via centrifugation (10 min; 1600×g). Plasma samples were stored at −80 °C for further detection. Patient data were recorded, with particular attention to Sequential Organ Failure Assessment (SOFA) scores within the first 24 h and 28‐day mortality (Tables S1 and S2, Supporting Information). Informed consent was obtained from the patients or their families, and the study was approved by the research ethics committee of the Third Xiangya Hospital of Central South University (2018‐S178). Plasma levels of IL1R2 in the septic patients were measured using the human IL1R2 DuoSet ELISA Kit (CatLog No. DY263, R&D Systems Co. USA) following the manufacturer's protocol. Due to the unbalanced sample sizes between groups, we performed a post‐hoc power analysis using G*Power software based on the one‐way ANOVA test in our study. This high level of power indicates a very low probability of Type II error, meaning that our study was highly capable of detecting true differences between the groups despite the unbalanced sample sizes.

### Analysis of the Retrospective Single‐Cell RNA Sequencing Data for IL1R2 Expression in Human Peripheral Immune Cells

We downloaded the dataset (GSE175453) from the gene expression omnibus (GEO) database (http://www.ncbi.nlm.nih.gov/geo),^[^ ^32^ ^]^ and analyzed it using by Cellenics platform (https://www.biomage.net/), an open‐source tool for analyzing single‐cell RNA sequencing (scRNA‐seq) datasets. The GSE175453 dataset includes gene expression profiles derived from whole blood myeloid‐enriched and Ficoll‐enriched peripheral blood mononuclear cells from four septic patients and four healthy subjects, utilizing Cellular Indexing of Transcriptomes and Epitopes by Sequencing (CITE‐seq). Prior to uploading to the GEO database, we performed barcode filtering and quality control on the dataset. Different immune cell clusters were annotated based on well‐known marker genes. Neutrophils were identified by co‐expressing FCGR3B, CEACAM8, s100a8; classic monocytes were identified by co‐expressing CD14, LYZ, CCR2; non‐classic monocytes were identified by co‐expressing FCGR3A, CD14(low), MS4A7; plasma B cells were identified by co‐expressing PRDM1 and XBP1, but without MS4A1(CD20); naïve CD4+ T cells were identified by co‐expressing CD4, CD3D, CCR7, and TCF7.

### Flow Cytometry Analysis of Monocytes in Septic Patients

Peripheral blood samples were collected from septic patients and healthy controls. For sample preparation, 100 µL of peripheral blood was lysed with red blood cell lysis buffer. After centrifugation at 1500×g for 5 min, the supernatant was discarded, and the pellet was resuspended in 100 µL of FACS buffer. Cells were stained with the following fluorescently labeled antibodies: APC/Cy7 anti‐human CD11b (Cat. No. 301342, Biolegend, San Diego, CA), FITC anti‐human CD14 (Cat. No. 301804, Biolegend), APC anti‐human CD16 (Cat. No. 302012, Biolegend), and PE anti‐human IL‐1R2 (Cat. No. 34141, Invitrogen). Staining was performed in the dark at room temperature for 30 min. Unstained cells were used for voltage calibration, while single‐stained cells were used for compensation. Data acquisition was performed on a BD flow cytometer, collecting 50000 events per sample, and analysis was conducted using FlowJo software (Tree Star, Ashland, OR). FSC‐A/SCC‐A gating strategy was used to apply to exclude debris and cell clumps. Classical monocytes (CD11b⁺CD14⁺CD16^−^) and non‐classical monocytes (CD11b⁺CD14^−^CD16⁺) were identified using FITC/APC gating. Finally, the mean fluorescence intensity (MFI) of PE‐IL1R2 was measured on both monocyte subsets in septic patients and healthy controls.

### Animal Studies

Male wild‐type (WT) C57BL/6J mice, age 6–8 weeks, were purchased from Hunan Slake Jingda Experimental Animals Co., Ltd (Hunan, China) and Charles River Laboratories (Wilmington, MA, USA). IL1R2 knockout (IL1R2^−/−^) mice on a C57BL/6 background were generated at Jiangsu Jicui Yaokang Biotechnology Co., Ltd. (Nanjing, China) using CRISPR/Cas9‐mediated transfection of guide RNA (gRNA). To validate the knockout strain, genomic PCR genotyping was performed (Figure S1, Supporting Information). All animals were housed in a 12 h dark/light cycle (25 ± 2 °C) under specific pathogen‐free (SPF) conditions, with ad libitum access to food and water. Experimental and control animals were co‐housed. All animal experiments were performed in compliance with the National Institutes of Health guidelines for the Care and Use of Laboratory Animals. The experiments were approved by the Institutional Animal Care and Use Committee of The Feinstein Institutes for Medical Research (2022‐015) and Central South University (2018sydw0344).

### Sepsis Induction and Treatment Protocol

Sepsis was induced in C57BL/6J mice by cecal ligation and puncture (CLP) as previously described.^[^ ^33^ ^]^ In brief, mice were anesthetized with isoflurane and placed in the supine position. A 1 cm midline laparotomy was created in the abdomen of mice to expose the abdominal cavity. The cecum was ligated and punctured with a 22‐G needle. A consistent and small amount of cecal content was extruded, and the cecum was then returned to the peritoneal cavity. Following abdominal closure, resuscitation was facilitated through subcutaneous (s.c.) injection of 500 µL of normal saline. For 20 h experiments, animals were not injected with antibiotics; however, for survival studies, animals were given 0.5 mg kg^−1^ body weight imipenem via s.c. Injection of 100 µL saline once, at the end of CLP surgery. All mice received a single s.c. Dose of 0.1 mg kg^−1^ buprenorphine immediately after CLP operation.

For the assessment of ENO1 inhibitor, AP‐III‐a4 (synonyms: Enoblock, Cat. No. HY‐15858, MCE), treatment based on the sepsis model, C57BL/6J mice were divided into four groups: Sham, Sham mice treated with Enoblock, CLP mice treated with vehicle, and CLP mice treated with Enoblock. Enoblock was given via retro‐orbital (RO) injection with the dose of 5 mg kg^−1^ at the time of surgery, once, immediately following abdominal closure. Vehicle groups received an equivalent volume of normal saline via RO injection. After 20 h, blood was collected by heart puncture. To ensure the reproducibility of experiments, all short‐term experiments were conducted at least three times. A 7‐day survival experiment was also conducted.

### Assessment of Organ Injury Markers

Serum levels of aspartate transaminase (AST), alanine transaminase (ALT), creatinine (Cre), and blood urea nitrogen (BUN) were determined using colorimetric enzymatic assays (Pointe Scientific, Canton, MI) according to the manufacturer's instructions.

### Assessment of Histological Lung Injury

The left lungs were embedded in paraffin and cut into sections 3 µm thick. Subsequently, morphological changes were observed with hematoxylin and eosin staining (H&E). Lung injury scores were evaluated in accordance with the most recent official American Thoracic Society workshop report on the features and measurements of experimental acute lung injury in animals.^[^ ^34^ ^]^ In summary, the severity of lung injury was assessed from 0 to 4, based on four independent domains: Histological evidence of tissue injury, alteration of the alveolar‐capillary barrier, presence of an inflammatory response, and evidence of physiological dysfunction. A score of 0 means no damage; l, < 25% damage; 2, 25% to 50% damage; 3, 50% to 75% damage, and 4, > 75% damage. The lung injury score was independently assessed by three pathologists who were blinded to the experimental conditions. The final score was determined by averaging the scores provided by each pathologist.

### Isolation of Mouse Peritoneal Macrophages

A method from the previous protocol was used for the isolation of mouse peritoneal macrophages.^[^ ^33^ ^]^ Briefly, mice were first injected intraperitoneally with thioglycolate broth to elicit an inflammatory response. Following a standard incubation period, the peritoneal cavity of mice was gently washed with phosphate‐buffered saline (PBS, Invitrogen) supplemented with 2% fetal bovine serum (FBS, Invitrogen). The peritoneal exudate cells were suspended in RPMI‐1640 supplemented with 10% FBS and 100 U mL^−1^ penicillin and seeded into 6‐well tissue culture plates at a density of 1×10^6^ cells/well and cultured for 4 h at 37 °C to allow them to adhere to the substrate. Non‐adherent cells were removed by gently washing with PBS. At this time, the adhered cells were macrophages and ready for experiments. The identity and purity of the resulting adherent macrophage population were confirmed by flow cytometry, validating for F4/80⁺ and CD11b⁺ expression.

### Cell Culture and Stimulation

The human embryonic kidney 293 (HEK293) T cell lines were obtained from the American Type Culture Collection (Rockville, MD, USA). The immortalized bone marrow‐derived macrophages (iBMDMs) were generously provided by Dr. Ben Lu. Those cells were cultured in DMEM supplemented with 10% FBS (Umedium, Hefei, China), 100 U L^−1^ of penicillin, and 100 µg L^−1^ of streptomycin. To induce proptosis, primary murine peritoneal macrophages and iBMDMs were primed with lipopolysaccharide (LPS, 1 µg mL^−1^) for 3 h followed by stimulation with ATP (5 mM, 0.5 h) or nigericin (5 µM, 1 h). To assess the role of GSDMD inhibition in IL1R2 release, iBMDMs were treated with or without disulfiram (5 µg mL^−1^). To examine the effect of ENO1 on macrophages, primary murine peritoneal macrophages and iBMDMs were treated with or without ENO1 inhibitor, Enoblock at indicated doses. To evaluate the impact of IL1R2 on nonclassical pyroptosis, cells were primed with LPS (1 µg mL^−1^) for 6 h, followed by LPS transfection (5 µg mL^−1^) for an additional 6 h.

### siRNA, Plasmids, and Transfection

For iBMDMs, siRNA was transfected using riboFECTTM CP Transfection Kit (Cat. No. C10511‐05, Ruibo Biotechnology Co., Ltd, Guangzhou, China) following the manufacturer's instructions. For IL1R2 or ENO1 siRNA transduction, iBMDMs were transfected with 100 nM IL1R2 siRNA (si‐IL1R2) or ENO1 siRNA (si‐ENO1), or negative control siRNA (NC, Control) using riboFECTTM CP reagent 48 h before the macrophage stimulation assay. The efficacy of the siRNA silencing was verified by detecting IL1R2 and ENO1 expressions by Western blotting. The following siRNA targeting sequences were used: IL1R2‐siRNA: CGCCTATTGATATCCAACA; Eno1‐siRNA: CCTTAAGGCTCTCCTCGGT.

### Overexpression of IL1R2 in iBMDM Cells

For IL1R2 overexpression, a lentiviral vector containing IL1R2 (NM_001360800) (GV341, Ubi‐IL1R2‐3FLAG‐SV40‐puromycin) and a control lentiviral vector were constructed (Genechem, Shanghai, China). The iBMDMs were seeded on a 6‐well plate in complete DMEM media, and lentivirus and HitransP were used according to the manufacturer's instructions. Following 48 h incubation period, the cells were screened by culture with 5 µg mL^−1^ puromycin, and individual colonies were obtained through serial dilution and amplification. Concurrently, cells were harvested, and IL1R2 expression was assessed by qPCR or Western blotting.

### Identification of IL1R2 Interacting Proteins by Mass Spectrometry

IL1R2‐overexpressing iBMDMs were treated with LPS (1 µg mL^−1^) for 3 h, followed by stimulation with ATP (5 mM, 0.5 h). Subsequently, the cells were lysed in NP‐40 buffer containing a protease inhibitor cocktail and kept on ice for 30 min. Cell extracts were subjected to centrifugation at 14000×g for 20 min at 4 °C, after which the supernatants were immunoprecipitated with anti‐Flag antibody (Cat. No. AE005, ABclonal) and then dissolved in 1 × SDS loading buffer. Prior to SDS‐PAGE, samples were boiled for 10 min at 95 °C. The proteins in the gel from different lanes were subjected to Liquid Chromatography Mass Spectrometry (LC‐MS/MS) analysis at Jingjie PTM Biolab Co., Ltd. (Hangzhou, China). In brief, the peptides were analyzed using Q ExactiveTM Plus mass spectrometry (Thermo Scientific) coupled to an ekspert EASY‐nLC 1000 (Thermo Scientific). The resulting MS/MS data were processed using Proteome Discoverer 2.4. The mass error was set to 10 ppm for precursor ions and 0.02 Da for fragmentations. Carbamidomethylation of cysteine residues was designated as a fixed modification, whereas oxidation of methionine residues was classified as a variable modification. The peptide confidence was set too high, and the peptide ion score was set to a value greater than 20.

### Enzyme‐Linked Immunosorbent Assay (ELISA)

Blood was collected by heart puncture, centrifuged at 1500×g, 4 °C for 15 min, and the plasma was separated to assess the cytokine levels using ELISA kits. TNFα (Cat. No. 558534, Biosciences), IL6 (Cat. No. 555240, BD Biosciences), IL1α (Cat. No. Cat. No. 88‐5019‐88, Invitrogen) and IL1β (Cat. No. 375912‐002, Invitrogen) levels in the plasma and cell culture supernatants were measured by ELISA according to the manufacturer's instructions.

### Lactate Dehydrogenase (LDH) Assay

iBMDMs or primary peritoneal macrophages were seeded in 96‐well plates. Cells were stimulated in Opti‐MEM medium (Invitrogen). At the end of incubations, cell culture supernatants were collected and cleared by centrifugation at 400×g for 10 min. LDH assays of cell supernatants were performed by CyQUANTTM LDH Cytotoxicity Assay kit (Cat. No. 2298167, Invitrogen) according to the manufacturer's protocol. The absorbance was measured at 490 nm and 680 nm using a BioTek Synergy Neo2 multi‐mode reader (BioTek Instruments, Inc.). Percentages of cytotoxicity were calculated by using the following formula: % of cytotoxicity = (compound‐treated LDH activity – spontaneous LDH activity) / (maximum LDH activity – spontaneous LDH activity) × 100.

### Immunofluorescence Assay

Harvested PerC macrophages were washed with cold PBS, then fixed with 4% formaldehyde for 15 min at room temperature (RT) and permeabilized with 0.1% Triton‐100 in PBS for 10 min. After washing with PBS, the cells were blocked with PBS/1% BSA for 30 min at RT. Subsequently, cells were incubated with primary antibody against enolase‐1 (dilution 1:200; Cat. Ab227978, Abcam,) and IL1R2 (dilution 1:200; Cat. No. AF563, R&D systems) overnight at 4 °C. Goat anti‐rabbit IgG (dilution 1:250; Cat. No. A21244, Invitrogen) and donkey anti‐goat IgG (dilution 1:250; Cat. No. A11058, Invitrogen) were served as secondary antibodies. Hoechst 33342 (Cat. No. R37605, Invitrogen) was used as a DNA dye. Cells were visualized using an Axio Observer Z1/7 equipped with a Zeiss LSM880A confocal microscopy system. The z‐stack images of cells were acquired with a Plan‐Apochromat 63x/1.40 Oil DIC M27 objective lens. SR‐4Y fast acquisition mode of Airyscan and 4× averaging was used. The images obtained by the confocal microscope were merged and combined by FIJI Image J.

### Western Blotting

After treatment, cell culture supernatants were collected and cleared of debris by centrifugation at 400×g for 10 min. Supernatants were precipitated with 10% trichloroacetic acid (TCA, Cat. No. BP555‐250, Fisher Scientific) on ice and washed with acetone. Protein precipitates were dissolved with 1× Tris buffer saline and 4× LDS sample buffer. Proteins were denatured by being heated for 5 min at 95 °C. iBMDMs or primary peritoneal macrophages were washed with PBS. Whole cell proteins were extracted and protein concentration in cell lysates was measured using the protein assay kit (Cat. No. 5000002, Bio‐Rad). Proteins were separated using NuPAGE 4%–12% Bis‐Tris gels (Invitrogen), transferred to PVDF membranes, and immunoblotted with the antibodies indicated in the figure legends according to the manufacturer's recommendations. Antibodies used in this study are: Gasdermin D (Cat. No. 39754S, 1:1000, Cell Signaling Technology), cleaved GSDMD (Cat. No. 10137S, 1:1000, Cell Signaling Technology or Cat. No. ab209845, 1:1000, Abcam), caspase1(Cat. No. 24232S, 1:1000, Cell Signaling Technology), cleaved Caspase1 (Cat. No. 89332S, 1:1000, Cell Signaling Technology), caspase11 (Cat. No. NB120‐10454, 1:1000, Novus), enolase 1 (Cat. No. MA5‐32756, 1:1000, Invitrogen or Cat. No. ab227978, 1:1000, Abcam), IL1R2 (Cat. No.AF563, 1:1000, R&D systems), IL1β (Cat. No. AF‐401‐SP, 1:1000, R&D systems), Flag (Cat. No. AE005 and AE063, 1:1000, ABclonal) and β‐actin (Cat. No. A5441, 1:10000 Sigma‐Aldrich) were used in indicated experiments. After incubation of the blots with primary antibodies, membranes were washed 3 times, and then the blots were subsequently incubated with the corresponding fluorescent secondary antibody (LI‐COR) or incubated with an HRP‐conjugated secondary antibody. Bands were detected by using the Odyssey FC Dual‐Mode Imaging system 2800 (LI‐COR) or visualized with ECL substrate (Bio‐Rad). Clarity Max Western ECL substrates (cat. 1705062, Bio‐Rad, Inc., Hercules, CA, USA) were used to perform Chemiluminescence enhancement.

### Co‐Immunoprecipitation (Co‐IP) Assay

To confirm the interaction between IL1R2 and ENO1, iBMDMs and 293T cells were lysed in NP‐40 buffer supplemented with 0.1 mM PMSF and a proteinase inhibitor cocktail and maintained on ice for 30 min. After centrifugation of the homogenate at 14000×g for 20 min at 4 °C, the protein concentration in the supernatants was measured using a bicinchoninic acid assay, and a portion of the supernatants was reserved as input for immunoblot analysis. An equal amount (300 µg) of the supernatants was incubated with specific Abs or IgG (1:50 dilution) and rotated at 4 °C overnight. The next day, protein A/G magnetic beads (30 µL, Cat. HY‐K0202, MCE) were added to the mixture, followed by 3 h rotation at 4 °C. After that, the immunoprecipitants were washed four times with NP‐40 buffer and boiled at 95 °C for 10 min in 1× SDS loading buffer for immunoblot analysis.

### Seahorse Assay

The extracellular acidification rate (ECAR) and oxygen consumption rate (OCR) were analyzed with a Seahorse XFp Analyzer instrument (Seahorse Bioscience, Billerica, MA) in WT and IL1R2^−/−^ peritoneal macrophages (PMs) as a measure of the glycolytic rate and OXPHOS, respectively. PMs were seeded overnight at a density of 1×10^5^ cells/well in wells B‐G with RPMI‐1640 growth medium (wells A and H contained media only) and stimulated with LPS (1 µg mL^−1^) or Enoblock (10 µM). The OCR was measured using an XFp Cell Mito Stress Test Kit (Cat. No. 103010–100, Agilent). Prior to starting the assay, the sensor cartridges were hydrated in Agilent Seahorse XF Calibrant at 37 °C in a non‐CO_2_ incubator overnight. The next day, cell culture growth medium was replaced with Seahorse XF RPMI medium (pH 7.4) supplemented with 1 mM pyruvate, 2 mM glutamine, and 10 mM glucose, and then the cell culture miniplates were placed into a 37 °C non‐CO_2_ incubator for at least 1 h. Oligomycin (an ATPase inhibitor, 1.5 µM), FCCP (1 µM), and rotenone plus antimycin A (0.5 µM) were injected where indicated, and the OCR (pMoles O_2_/min) was measured in real time following the manufacturer's standard protocols. The ECAR was measured using an XFp Glycolysis Stress Test kit (Cat. No. 103017–100, Agilent). The preparation stage is analogous to that described above, except for the addition of 2 mM glutamine to the Seahorse XF assay medium (pH 7.4). Glucose (10 mM), oligomycin (1 µM), and 2‐DG (50 mM) were injected at the appropriate times. OCR and ECAR data were normalized to cell count after the Seahorse run.

### Enolase Activity Assay

Enolase Activity Assay Kit (Cat. MAK178, Sigma‒Aldrich) was used for the purpose of conducting an assay to determine the activity of ENO1. Primary macrophages were seeded into 12‐well plates and pretreated with or without Enoblock (10 µM, Cat. No. HY‐15858, MCE). After washing with PBS, cells (1×10^6^) were homogenized in 100 µL of ice‐cold Enolase Assay Buffer and centrifuged at 10000×g for 5 min to remove insoluble substances. According to the manufacturer's instructions, the supernatants were extracted and determined by a coupled enzyme assay. One milliunit of enolase 1 is defined as the amount of enzyme capable of generating 1.0 nmol of H_2_O_2_ per minute at pH 7.2 and 25 °C.

### Propidium Iodide (PI) Staining

PI staining buffer was used to perform PI staining following the manufacturer's instructions. Cells were seeded into 24‐well plates and treated with appropriate stimuli. The assay was incubated for 10 min with the addition of PI dye (50 µg mL^−1^), and fluorescence and phase‐contrast images were acquired on multiple occasions. The percentage of cell death was the number of PI‐positive cells divided by the total number of cells.

### Homology Modeling of ENO1 and IL1R2

The amino acid sequences of mouse ENO1 and IL1R2 were retrieved from the Uniprot database. The models were generated using the Iterative Threading ASSEmbly Refinement (I‐TASSER) server based on templates identified by the threading approach to maximize the percentage identity, sequence coverage, and confidence. The docking of ENO1 and IL1R2 protein structure models was performed using the ATTRACT tool, which uses an approach of conformational flexibility of binding partners. The docking process includes pre‐calculation of potential energy on a grid, and then interactions are calculated by interpolation from nearest grid points. The protein‐protein interactions between ENO1 and IL1R2 were calculated using the PDBePISA tool, which calculated the protein‐protein interaction interface and thermodynamic properties of interaction.

### Surface Plasmon Resonance Assay

Surface plasmon resonance (SPR) assay was performed as described previously.^[^ ^33^ ^]^ To investigate the direct interaction between recombinant mouse ENO1 (rmENO1) and rmIL1R2, a SPR assay was performed using OpenSPR (Nicoya). rmENO1 (Cat. No. Eno1‐244 M) and rmIL1R2 (Cat. No. 563‐MR‐100) were sourced from Creative BioMart and R&D Systems, respectively. The assay was conducted by immobilizing rmIL1R2 on a high‐sensitivity carboxyl sensor and subsequently injecting rmENO1 as the analyte in a reaction buffer (50 mM Tris‐HCl, 150 mM NaCl, 3 mM EDTA, 0.05% P20, 0.5 mM CaCl_2_, pH 7.4). Initially, the carboxyl sensor was prepared by injecting 150 µL of 10 mM HCl, followed by a 150 µL injection of a 1:1 mixture of N‐ethyl‐N′‐[3‐diethylaminopropyl]‐carbodiimide (EDC) and N‐hydroxysuccinimide (NHS) to activate the sensor surface. Subsequently, 200 µL of 30 µg mL^−1^ rmIL1R2 in 10 mM sodium acetate (pH 5) was injected into flow cell channel 2 for immobilization. Remaining active sites on channels 1 and 2 were then deactivated with 150 µL of 1 M ethylenediamine (pH 8.5). Flow cell 1 served as a control to assess non‐specific binding. Binding analyses were carried out at a flow rate of 40 µL min^−1^ at 20 °C. Analyte concentrations ranging from 12.5 to 50 nM were injected into both flow cells, and real‐time interaction data were analyzed using TraceDrawer (Nicoya). Signals from the control channel (flow cell 1) were subtracted from the ligand‐coated channel (flow cell 2) for all samples. The data were globally fitted for a 1:1 binding model, yielding a dissociation constant (K_D_) of 1.99×10^−9^ M.

### Statistical Analysis

All statistical analyses were conducted using GraphPad Prism version 8.0 software (GraphPad Software, La Jolla, CA) or R 3.3.2 software. Prior to analysis, data were assessed for normal distribution and for outliers, which were removed if identified. The sample size (*n*) for each experimental group is indicated in the figure legends or relevant results sections. Data are shown as the mean ± standard error of mean (SEM). Comparisons between two groups were performed with a two‐tailed Student's t test. Comparisons between multiple groups were analyzed using one‐way or two‐way analysis of variance (ANOVA) followed by Tukey's multiple comparisons test. Survival rates were compared using Kaplan‐Meier survival plots and a log‐rank test. Significance was considered for *p* < 0.05 between study groups.

## Conflict of Interest

The authors declare no conflict of interest.

## Author Contributions

C.T. and H.M. contributed equally to this work and share the first authorship. M.A., H.C., H.Z., and P.W. contributed equally to this work and share the senior authorship. C.T., M.A., P.W., C.H., and H.Z. performed conceptualization. C.T., H.M., J.C., G.M., A.J., S.T., Y.L., M.L., and K.L. performed methodology. C.T., H.M., J.C., G.M., A.J., S.T., Y.L., M.L., and K.L. performed the investigation. C.T., H.C., K.W., X.X., M.A., P.W., C.H., and H.Z. performed visualization. P.W., M.A., C.H., and H.Z. performed funding acquisition. M.A., C.H., P.W., and H.Z. did project administration. K.W., X.X., M.A., P.W., C.H., and H.Z. performed supervision. C.T. and H.M. wrote the original draft. C.T., H.M., C.H., M.A., P.W., and H.Z. wrote, reviewed, and edited.

## Supporting information



Supporting Information

Supporting Information

## Data Availability

High‐resolution scans of all blots presented in the paper have been included as Data S2 – Source data to this manuscript. All other data are available from the corresponding author on request.
