# Supplementary material for: Critical Role of IL1R2‐ENO1 Interaction in Inhibiting Glycolysis‐Mediated Pyroptosis for Protection Against Lethal Sepsis
Source: Adv Sci (Weinh). 2025 Jul 24;12(39):e02297. doi: 10.1002/advs.202502297 (PMC12533375; doi:10.1002/advs.202502297)
Supplement: Supplementary file 1 — Supporting Information [file ADVS-12-e02297-s001.pptx]

## Slide 1
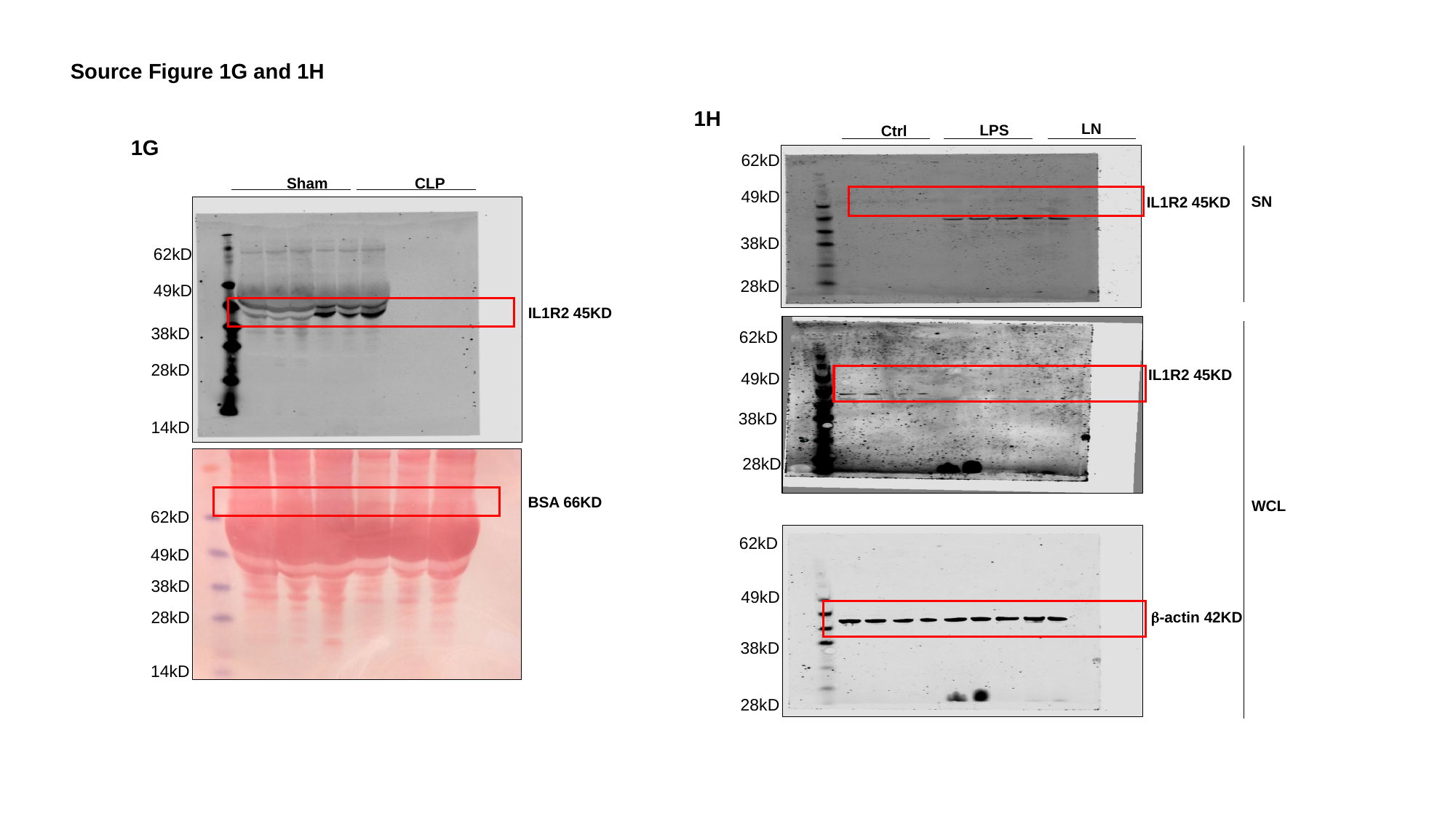

Source Figure 1G and 1H
1H
LN
LPS
Ctrl
1G
62kD
49kD
SN
IL1R2 45KD
38kD
28kD
62kD
IL1R2 45KD
49kD
38kD
28kD
WCL
62kD
49kD
β-actin 42KD
38kD
28kD
1G
Sham
CLP
62kD
49kD
IL1R2 45KD
38kD
28kD
14kD
BSA 66KD
62kD
49kD
38kD
28kD
14kD

## Slide 2
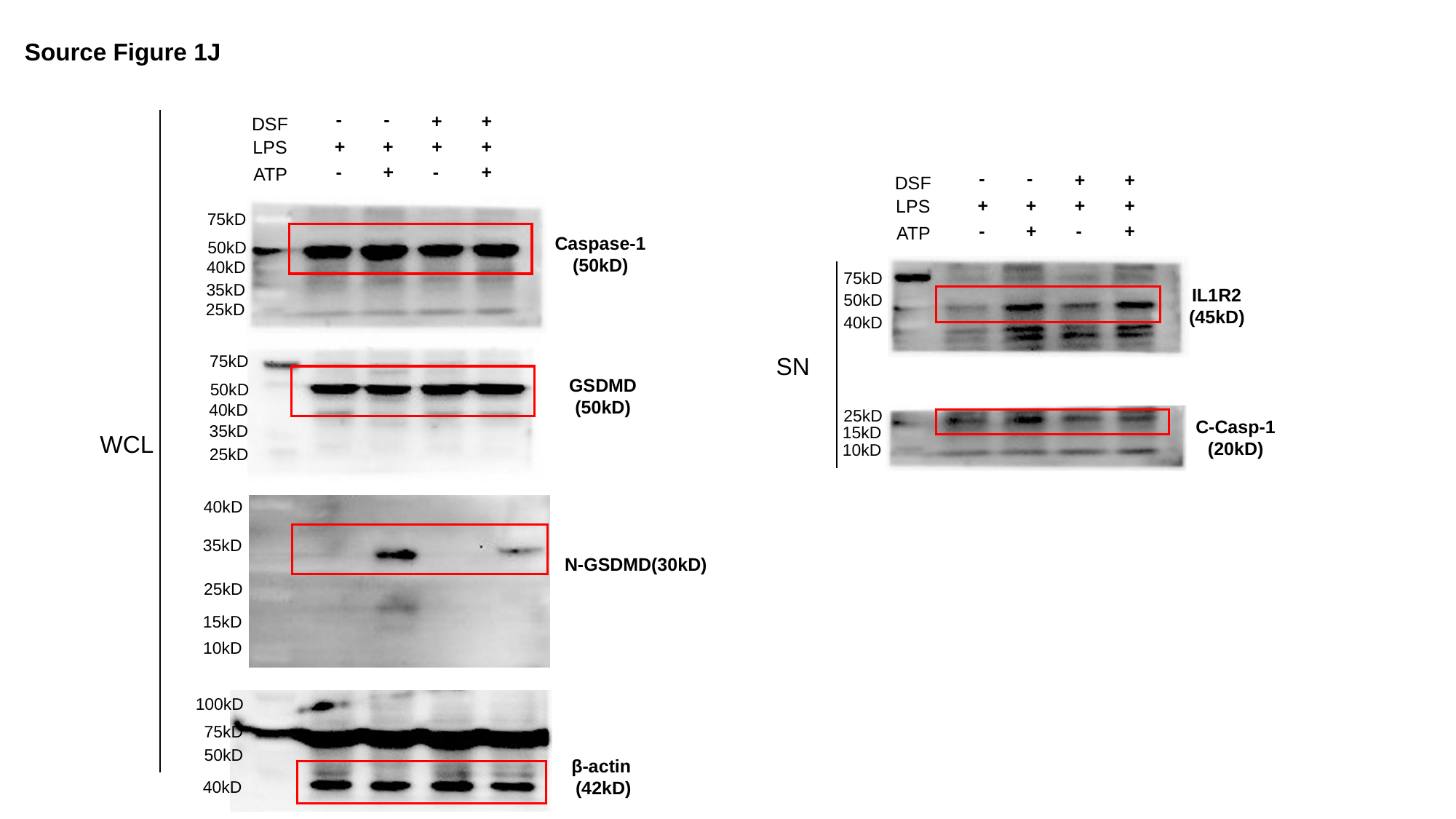

Source Figure 1J
-
-
+
+
DSF
+
+
+
+
LPS
-
+
-
+
ATP
-
-
+
+
DSF
+
+
+
+
LPS
-
+
-
+
ATP
75kD
Caspase-1
(50kD)
50kD
40kD
35kD
25kD
75kD
IL1R2
(45kD)
50kD
40kD
75kD
GSDMD
(50kD)
50kD
40kD
35kD
25kD
SN
25kD
C-Casp-1
(20kD)
15kD
10kD
WCL
40kD
35kD
N-GSDMD(30kD)
25kD
15kD
10kD
100kD
75kD
50kD
β-actin
(42kD)
40kD

## Slide 3
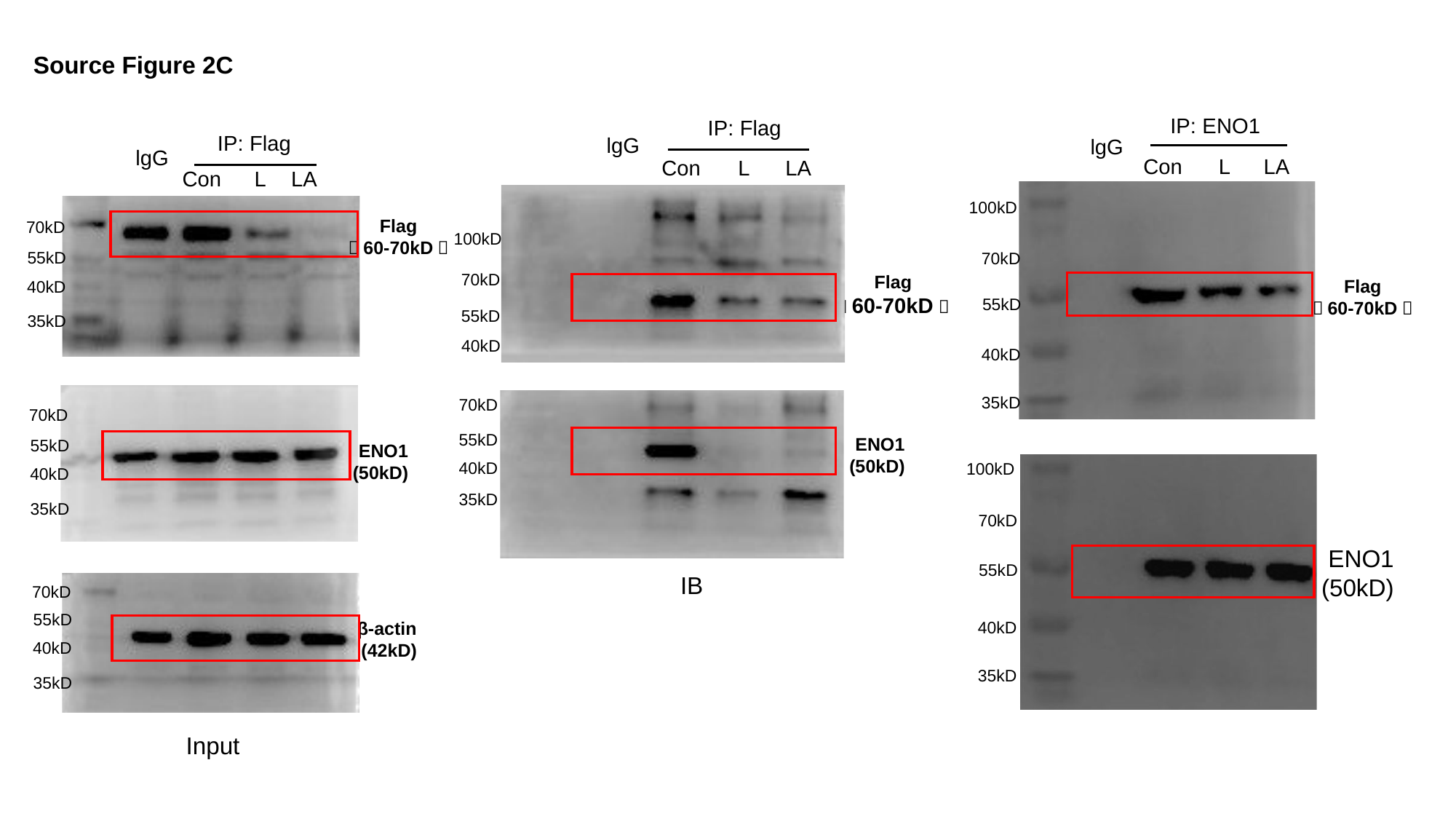

Source Figure 2C
IP: ENO1
lgG
L
LA
Con
100kD
70kD
Flag
（60-70kD）
55kD
40kD
35kD
100kD
70kD
ENO1 (50kD)
55kD
40kD
35kD
IP: Flag
lgG
L
LA
Con
100kD
70kD
Flag
（60-70kD）
55kD
40kD
70kD
55kD
ENO1
(50kD)
40kD
35kD
IB
IP: Flag
lgG
L
LA
Con
Flag
（60-70kD）
70kD
55kD
40kD
35kD
70kD
55kD
ENO1
(50kD)
40kD
35kD
70kD
55kD
β-actin
(42kD)
40kD
35kD
Input

## Slide 4
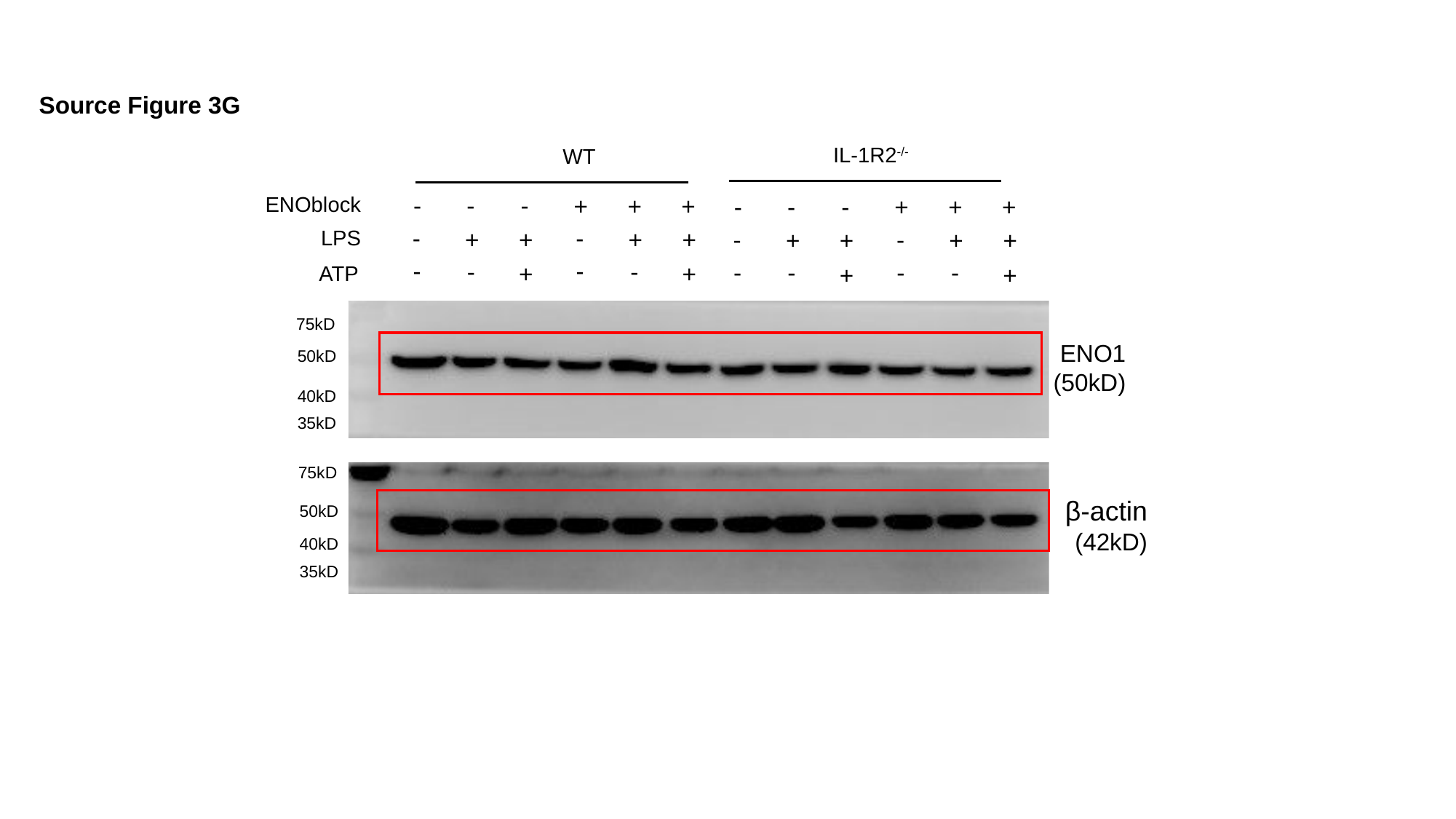

Source Figure 3G
IL-1R2-/-
WT
-
-
-
ENOblock
+
+
+
-
-
-
+
+
+
-
-
+
+
+
+
-
-
LPS
+
+
+
+
-
-
-
-
-
-
-
-
+
+
+
+
ATP
75kD
ENO1
(50kD)
50kD
40kD
35kD
75kD
β-actin
(42kD)
50kD
40kD
35kD

## Slide 5
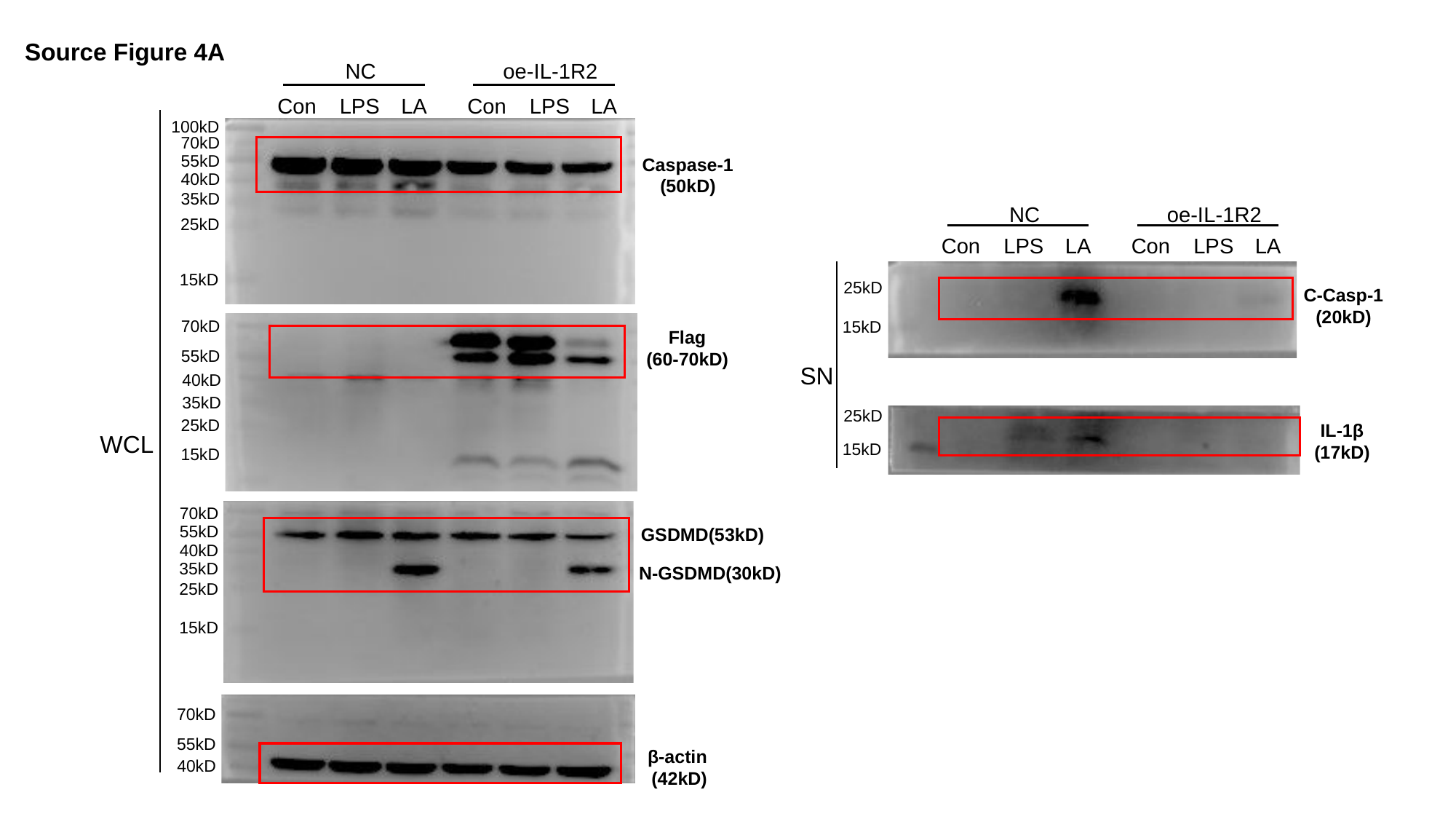

Source Figure 4A
NC
oe-IL-1R2
LPS
LA
LPS
LA
Con
Con
100kD
70kD
55kD
Caspase-1
(50kD)
40kD
35kD
25kD
15kD
NC
oe-IL-1R2
LPS
LA
LPS
LA
Con
Con
25kD
C-Casp-1
(20kD)
70kD
Flag
(60-70kD)
55kD
40kD
35kD
25kD
15kD
15kD
SN
25kD
IL-1β
(17kD)
WCL
15kD
70kD
55kD
GSDMD(53kD)
40kD
35kD
25kD
15kD
N-GSDMD(30kD)
70kD
55kD
β-actin
(42kD)
40kD

## Slide 6
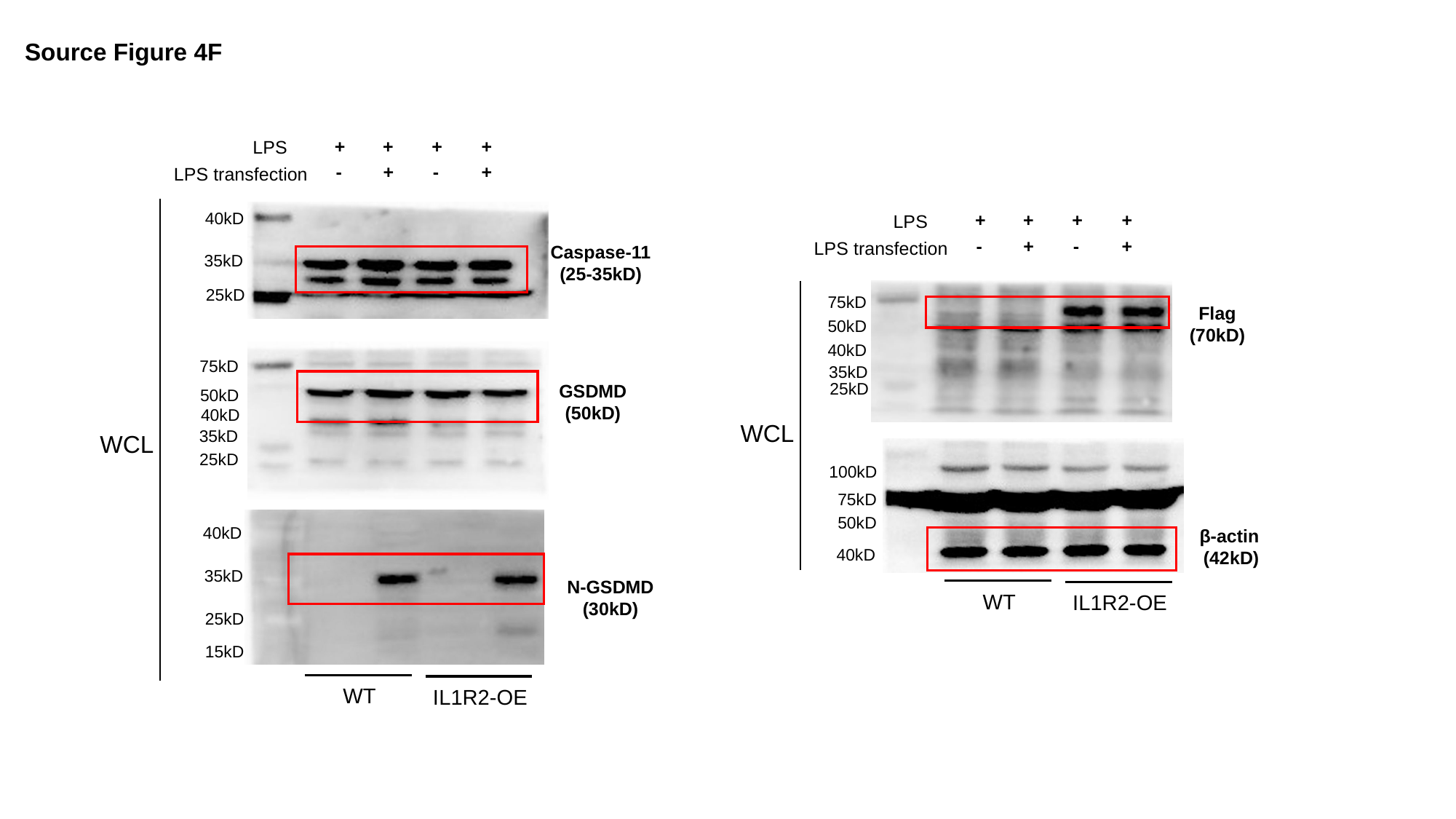

Source Figure 4F
+
+
+
+
LPS
-
+
-
+
LPS transfection
40kD
Caspase-11
(25-35kD)
35kD
25kD
+
+
+
+
LPS
-
+
-
+
LPS transfection
75kD
Flag
(70kD)
50kD
40kD
35kD
25kD
75kD
GSDMD
(50kD)
50kD
40kD
35kD
25kD
WCL
WCL
100kD
75kD
50kD
β-actin
(42kD)
40kD
40kD
35kD
N-GSDMD
(30kD)
25kD
15kD
WT
IL1R2-OE
WT
IL1R2-OE

## Slide 7
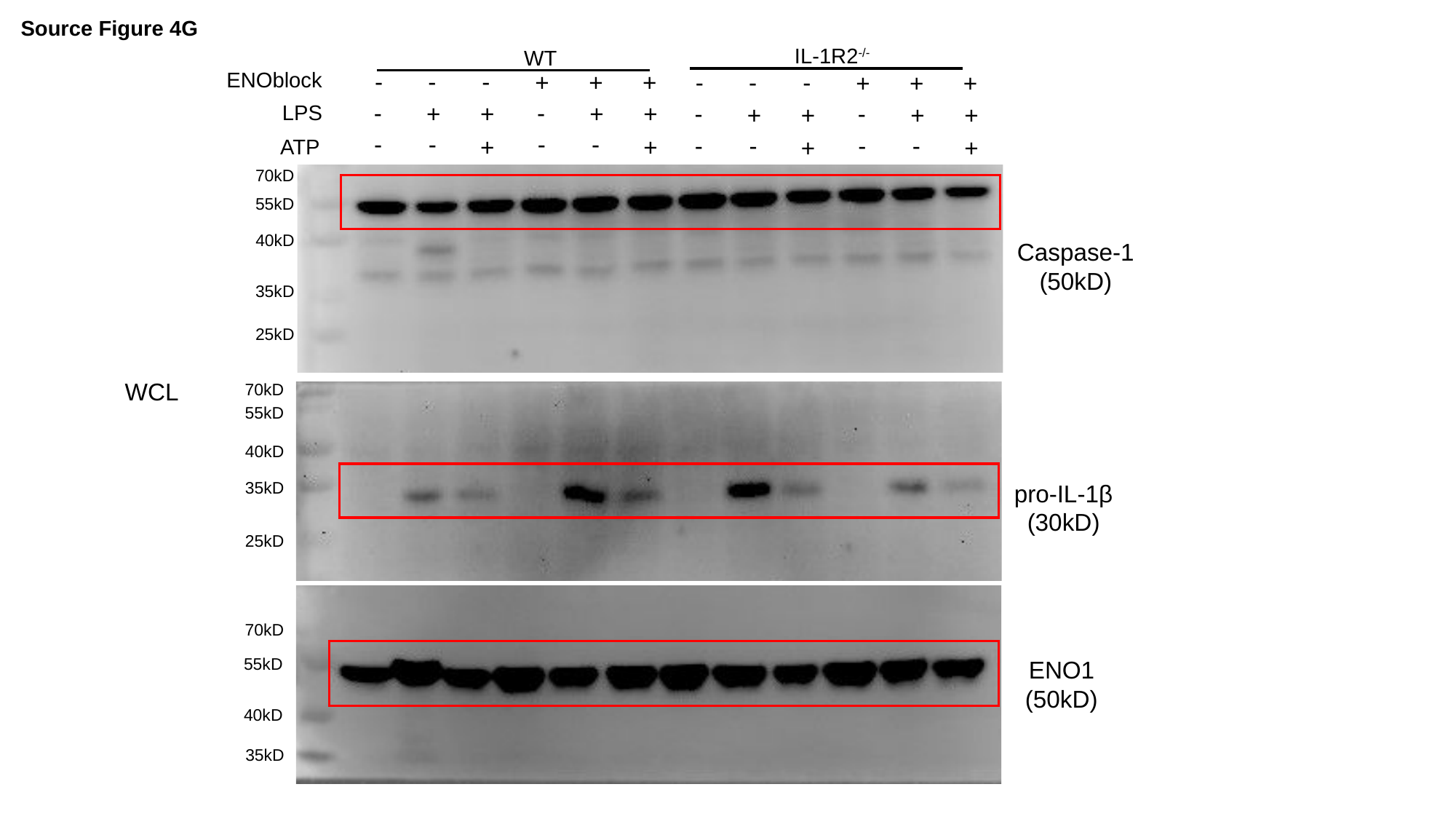

Source Figure 4G
IL-1R2-/-
WT
-
-
-
ENOblock
+
+
+
-
-
-
+
+
+
-
-
+
+
+
+
-
-
LPS
+
+
+
+
-
-
-
-
-
-
-
-
+
+
+
+
ATP
70kD
55kD
40kD
Caspase-1
(50kD)
35kD
25kD
WCL
70kD
55kD
40kD
35kD
pro-IL-1β
(30kD)
25kD
70kD
55kD
ENO1
(50kD)
40kD
35kD

## Slide 8
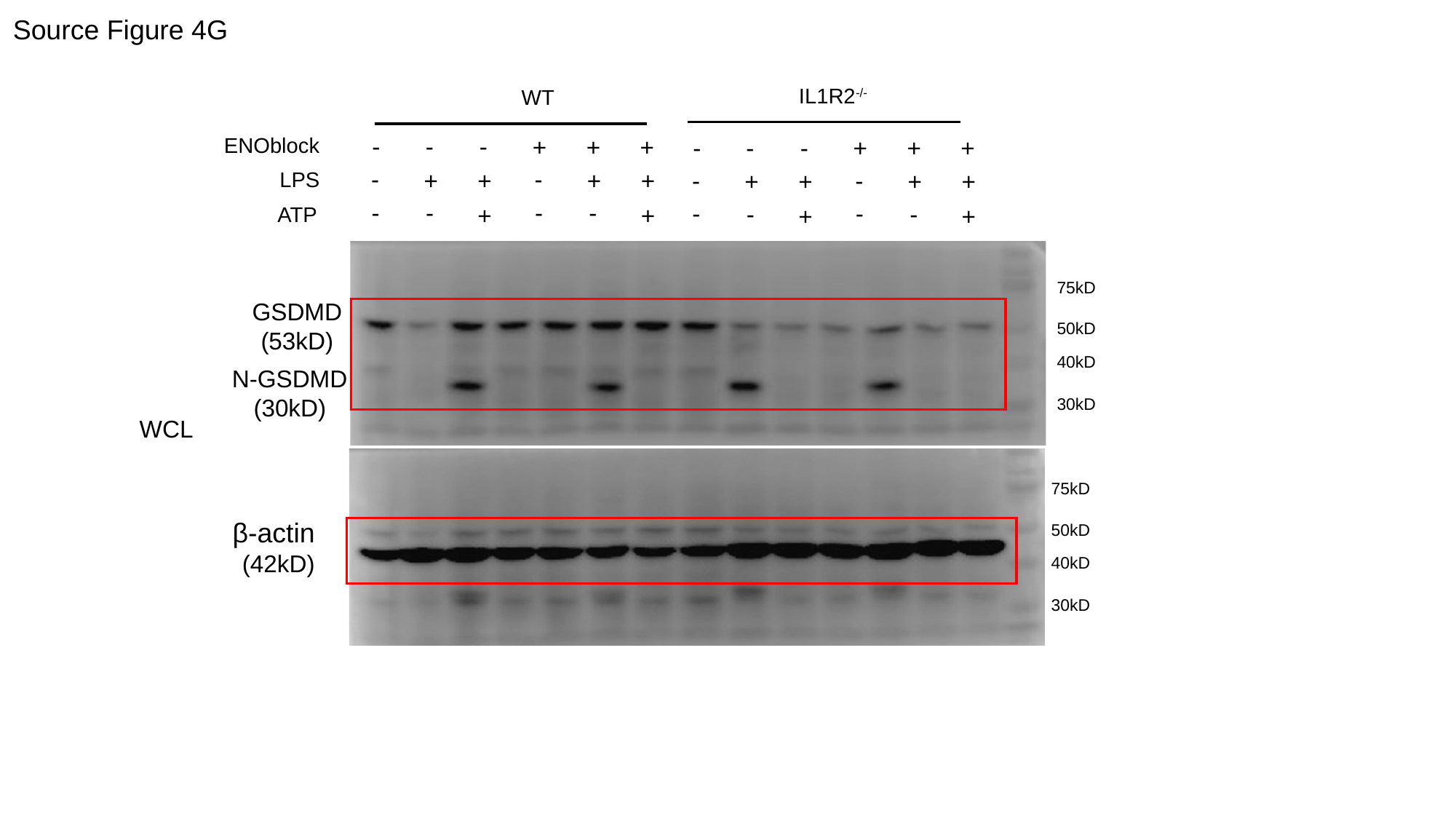

Source Figure 4G
IL1R2-/-
WT
-
-
-
ENOblock
+
+
+
-
-
-
+
+
+
-
-
+
+
+
+
-
-
LPS
+
+
+
+
-
-
-
-
-
-
-
-
+
+
+
+
ATP
75kD
GSDMD
(53kD)
50kD
40kD
30kD
N-GSDMD
(30kD)
WCL
75kD
β-actin
(42kD)
50kD
40kD
30kD

## Slide 9
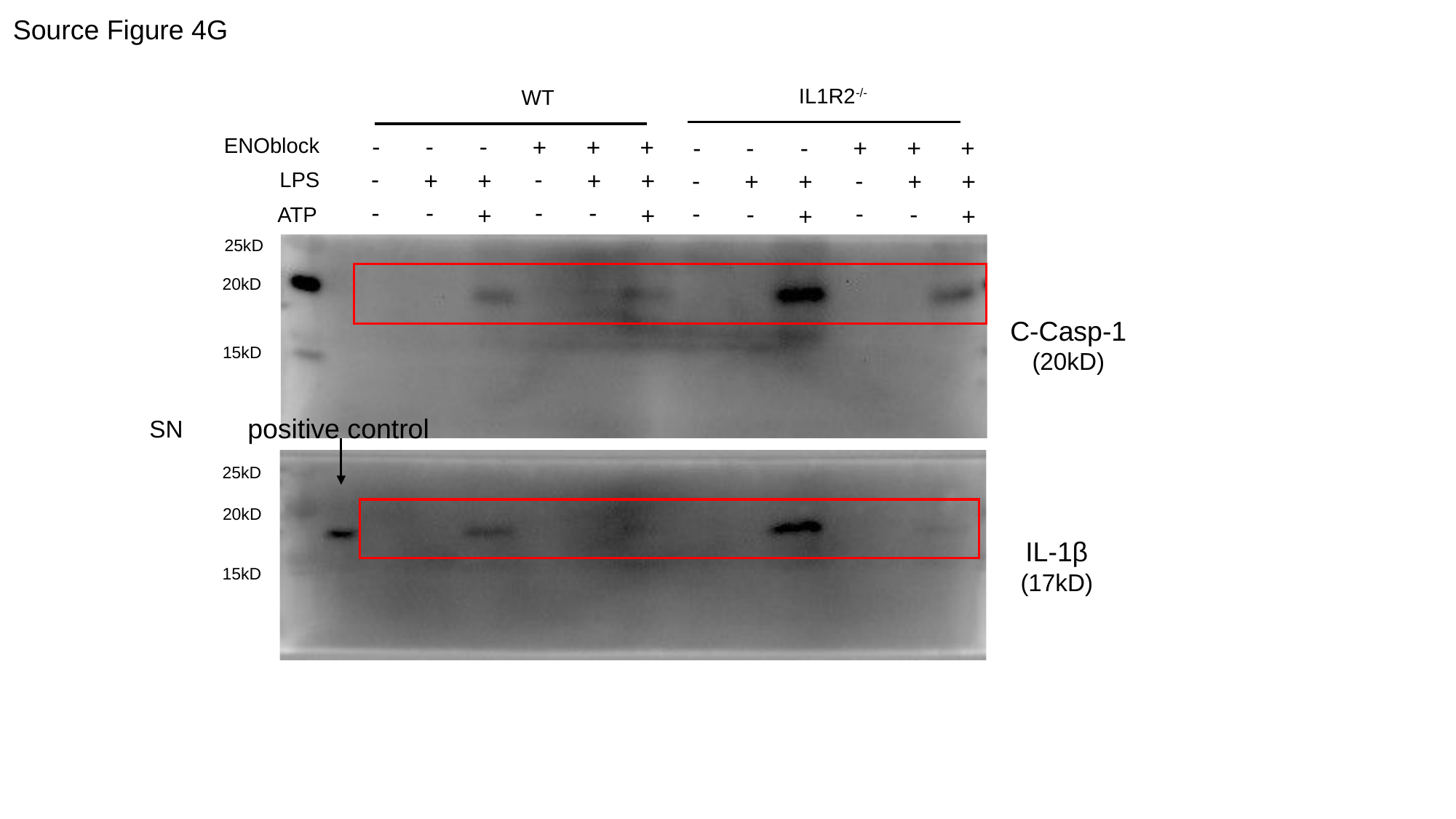

Source Figure 4G
IL1R2-/-
WT
-
-
-
ENOblock
+
+
+
-
-
-
+
+
+
-
-
+
+
+
+
-
-
LPS
+
+
+
+
-
-
-
-
-
-
-
-
+
+
+
+
ATP
25kD
20kD
C-Casp-1
(20kD)
15kD
positive control
SN
25kD
20kD
IL-1β
(17kD)
15kD

## Slide 10
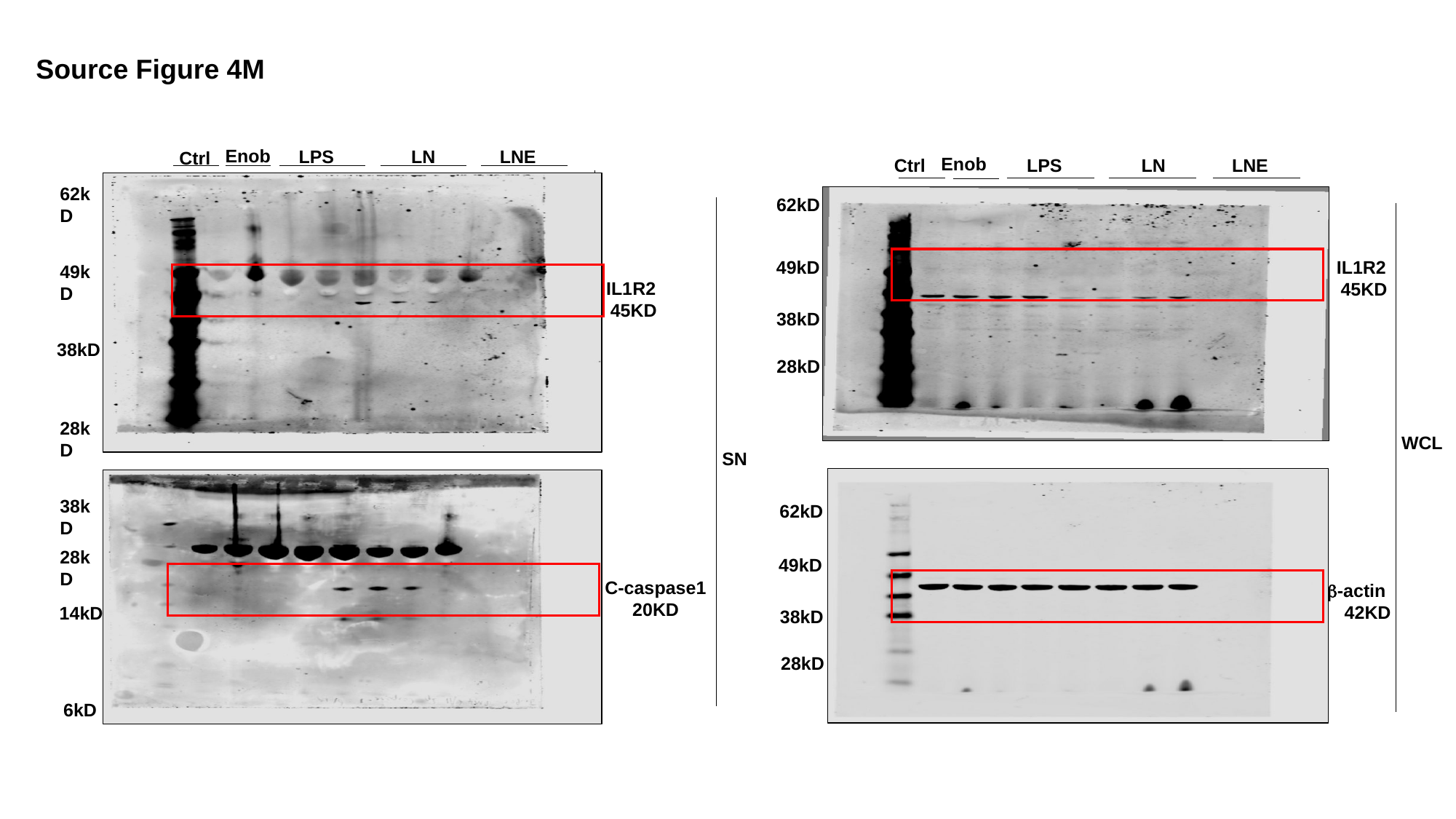

Source Figure 4M
Enob
LPS
LN
LNE
Ctrl
62kD
49kD
38kD
28kD
38kD
28kD
14kD
6kD
IL1R2
45KD
C-caspase1
20KD
SN
Enob
Ctrl
LPS
LN
LNE
62kD
49kD
38kD
28kD
WCL
62kD
49kD
38kD
28kD
IL1R2
45KD
β-actin
42KD

## Slide 11
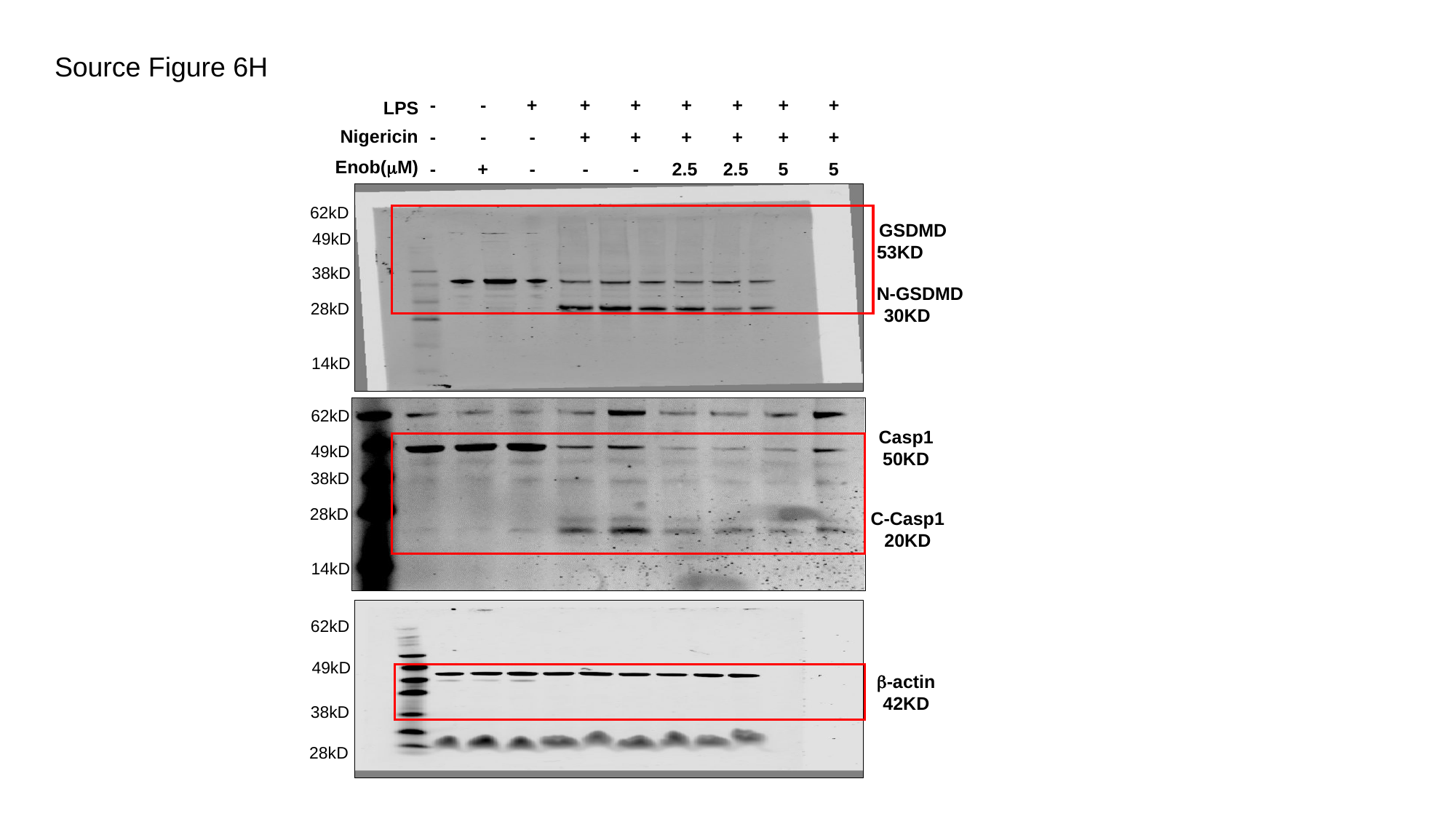

Source Figure 6H
-
-
+
+
+
+
+
+
+
LPS
-
-
-
+
+
+
+
+
+
-
+
-
-
-
2.5
2.5
5
5
62kD
 GSDMD
53KD
49kD
38kD
 N-GSDMD
30KD
28kD
14kD
62kD
Casp1
50KD
49kD
38kD
28kD
C-Casp1
20KD
14kD
62kD
49kD
β-actin
42KD
38kD
28kD
Nigericin
 Enob(μM)

## Slide 12
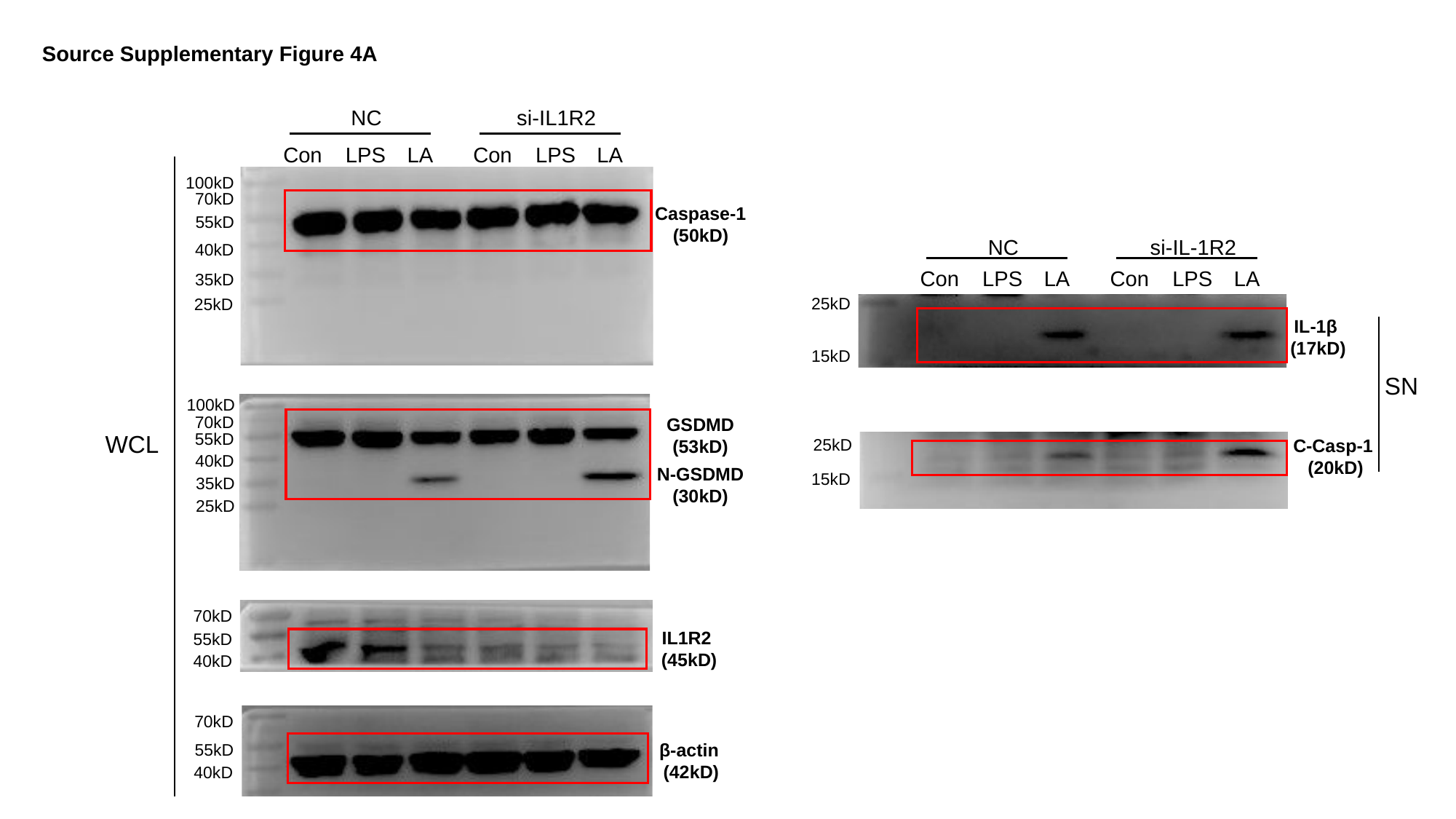

Source Supplementary Figure 4A
NC
si-IL1R2
LPS
LA
LPS
LA
Con
Con
100kD
70kD
Caspase-1
(50kD)
55kD
40kD
35kD
25kD
NC
si-IL-1R2
LPS
LA
LPS
LA
Con
Con
25kD
IL-1β
(17kD)
15kD
SN
100kD
70kD
GSDMD
(53kD)
55kD
40kD
35kD
25kD
WCL
25kD
C-Casp-1
 (20kD)
15kD
N-GSDMD
(30kD)
70kD
IL1R2
(45kD)
55kD
40kD
70kD
β-actin
(42kD)
55kD
40kD

## Slide 13
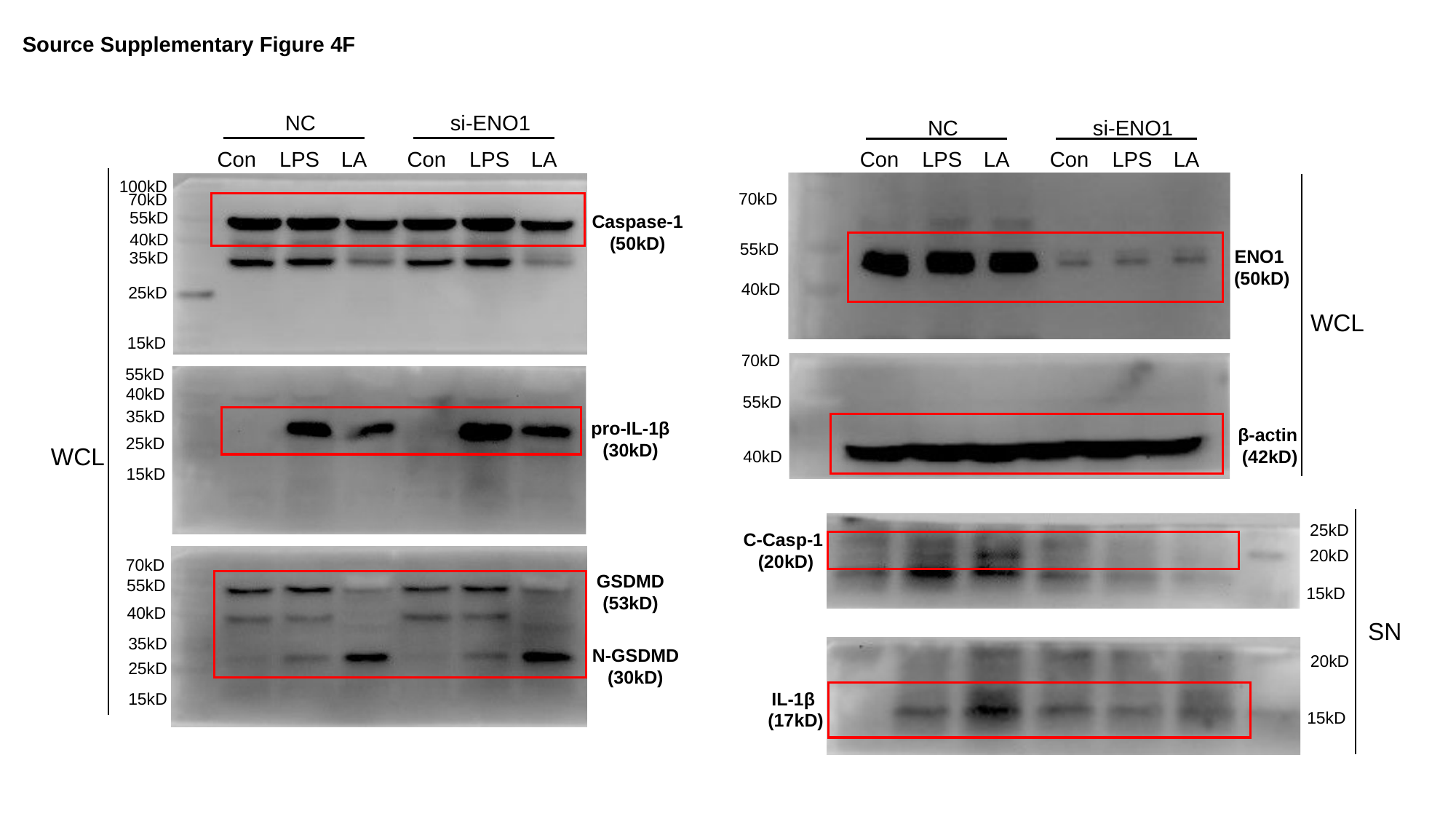

Source Supplementary Figure 4F
NC
si-ENO1
LPS
LA
LPS
LA
Con
Con
NC
si-ENO1
LPS
LA
LPS
LA
Con
Con
100kD
70kD
55kD
Caspase-1
(50kD)
40kD
35kD
25kD
15kD
70kD
55kD
ENO1
(50kD)
40kD
WCL
70kD
55kD
40kD
35kD
pro-IL-1β
(30kD)
25kD
15kD
55kD
β-actin
(42kD)
WCL
40kD
25kD
C-Casp-1
 (20kD)
20kD
15kD
70kD
GSDMD
(53kD)
55kD
40kD
35kD
25kD
15kD
SN
20kD
IL-1β
 (17kD)
15kD
N-GSDMD
(30kD)
