# Supplementary material for: Critical Role of IL1R2‐ENO1 Interaction in Inhibiting Glycolysis‐Mediated Pyroptosis for Protection Against Lethal Sepsis
Source: Adv Sci (Weinh). 2025 Jul 24;12(39):e02297. doi: 10.1002/advs.202502297 (PMC12533375; doi:10.1002/advs.202502297)
Supplement: Supplementary file 2 — Supporting Information [file ADVS-12-e02297-s002.pdf]

ADVANCED SCIENCE
Open Access

Supporting Information

for *Adv. Sci.*, DOI 10.1002/advs.202502297

Critical Role of IL1R2-ENO1 Interaction in Inhibiting Glycolysis-Mediated Pyroptosis for Protection Against Lethal Sepsis

Chuyi Tan, Han Ma, Jespar Chen, Gaifeng Ma, Alok Jha, Sipin Tan, Yaxi Zhu, Meidong Liu, Ke Liu, Xianzhong Xiao, Monowar Aziz, Huan Chen, Ping Wang and Huali Zhang**

Supplementary Data

Critical Role of IL1R2-ENO1 Interaction in Inhibiting Glycolysis-Mediated Pyroptosis for Protection Against Lethal Sepsis

Chuyi Tan[1,2,3#], Han Ma[2,3#], Jespar Chen[1], Gaifeng Ma[1], Alok Jha[1], Sipin Tan[2,3], Yaxi Zhu[2,3], Meidong Liu[2,3], Ke Liu[2,3], Xianzhong Xiao[2,3], Monowar Aziz[1,4*], Huan Chen[2,3*], Ping Wang[1,4*], Huali Zhang[2,3*]

[1]Center for Immunology and Inflammation, the Feinstein Institutes for Medical Research, Manhasset, New York, USA.
[2]Key Laboratory of Sepsis Translational Medicine of Hunan, Department of Pathophysiology, School of Basic Medicine Science, Central South University, Changsha, Hunan, P.R.China.
[3]National Medicine Functional Experimental Teaching Center, Central South University, Changsha, Hunan, P.R.China.
[4]Departments of Surgery and Molecular Medicine, Zucker School of Medicine at Hofstra/Northwell, Manhasset, New York, USA.

Supplementary Methods

Analysis of the retrospective single-cell RNA sequencing data for IL1R2 expression in the lung and liver of CLP induced septic mice

We download the dataset (GSE207651 and GSE279167) from the gene expression omnibus (GEO) database (http://www.ncbi.nlm.nih.gov/geo)[1, 2], and analyzed it by Cellenics platform (https://www.biomage.net/), an open-source tool for analyzing single-cell RNA sequencing (scRNA-seq) datasets. The GSE207651 dataset includes gene expression profiles derived from mouse lung tissue cells from isolated pooled from 3 sham and 4 mice with CLP induced sepsis. the Sham group, CLP group, and the CLP-48h group. The GSE279167 dataset includes gene expression profiles derived from mouse liver tissue cells from isolated pooled from 3 sham and 4 mice with CLP induced sepsis. Prior to uploading to the GEO database, barcode filtering and quality control on the dataset was performed. Immune cell clusters were annotated based on well-established marker genes.

Macrophage-specific IL1R2 knockdown mice

To construct mice with macrophage-specific knockdown of IL1R2 ($IL1R2^{CKD}$), $IL1R2^{CKD}$ adenovirus [GV684, pAAV-CD68p-EGFP-mir155*3 (mIL1R2)-WPRE-SV40 PolyA] and adenovirus negative control (NC) were purchased from Genechem (Shanghai, China). Adenovirus serotype 6 was the source and nature of adenoviruses. The $IL1R2^{CKD}$ and negative control Adenovirus were injected via the tail vein at a dose of 2×10^{11} viral PFU/mouse 4 weeks prior to the commencement of the animal experiments. The impact of macrophage-specific IL1R2 knockdown was evaluated through flow cytometry (**Supplementary Figure 5**).

Assessment of IL-1R2^{CKD} mice by Flow cytometry

Bone marrow-derived cells were extracted from WT and IL-1R2^{CKD} mice and evaluated by flow cytometry. A total of 1×10^6 bone marrow-derived cells in 100 μL FACS buffer were stained with APC anti-mouse/human CD11b Ab (Cat. No. 101211, Biolegend, San Diego, CA), PE-Cy7 anti-mouse CD86 Ab (Cat. No. 105029, Biolegend), PerCP-Cy5.5 anti-mouse Ly6G Ab (Cat. No. 127616, Biolegend) and PE anti-mouse CD121b (IL1R2) Ab (Cat. No. 554450, BD Biosciences, San Jose, CA) at room temperature (RT) in the dark for 30 min. The unstained cells were used to adjust the voltage setting, while single-stained cells were used for single-color compensation. 50,000 events were collected using a BD flow cytometer

and the data were analyzed using FlowJo software (Tree Star, Ashland, OR). FSC-A/SCC-A gating strategy were used to distinguish cells from fragments and clumps. FITC/PE-Cy7 and FITC/PerCP-Cy5.5 gating strategies were used to select macrophages and neutrophils, respectively. Subsequently, the mean fluorescence intensity (MFI) of PE-IL1R2 on CD11b^{+}CD86^{+} macrophages and CD11b^{+}Ly6G^{+} neutrophils from the bone marrow of WT and IL-1R2^{CKD} mice were separately measured to confirm the impact of conditional knockdown.

Quantitative real-time PCR

RNA Fast 200 kit (FASTAGEN) was used to extract total RNA from lung tissues or peritoneal macrophages of WT and IL1R2$^{-/-}$ mice according to the manufacturer's instructions. Complementary DNA was synthesized by using the Evo M-MLV RT Mix Kit (Cat. No. AG11728, Accurate Biology). Quantitative PCR was performed using SYBR® Green Premix (Cat. No. AG11701, Accurate Biology) and CFX96 instruments (Bio-Rad Laboratories, CA, USA) according to the manufacturer's protocols. Data were normalized to β-actin expression, and the relative gene expression changes were calculated using the $2^{(-\Delta\Delta CT)}$ method. The sequence of primers used in the present study were listed in **Supplementary table 5.**

Supplementary references

1. Wang, F., et al., Integrating bulk and single-cell sequencing reveals the phenotype-associated cell subpopulations in sepsis-induced acute lung injury. Front Immunol, 2022. **13**: p. 981784.
2. Lavarti, R., et al., Senescence landscape in the liver following sepsis and senolytics as potential therapeutics. Aging Cell, 2024: p. e14354.

Supplementary Figure 1

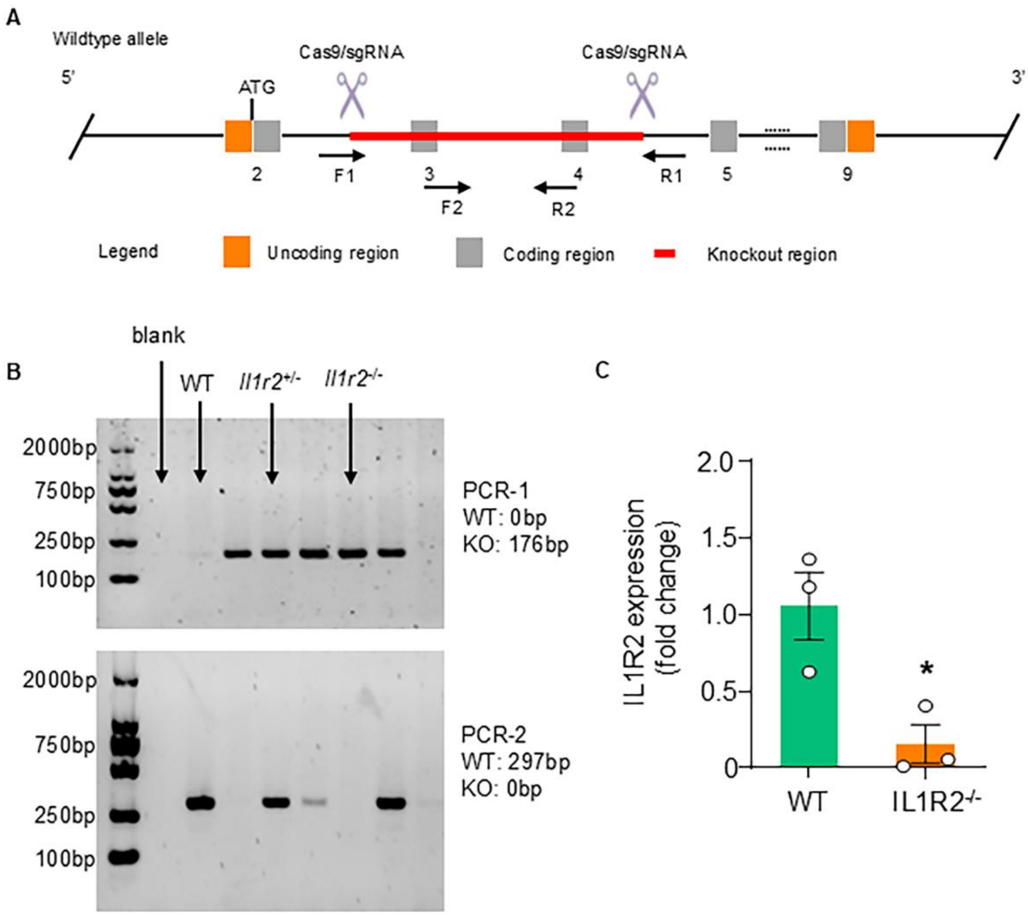

Supplementary Figure 1: Strategy for creating IL1R2 knockout mice. (A) Genotyping strategy for creating IL1R2 knockout mice. **(B, C)** PCR screening and RT-PCR analysis of F1 pups for genotyping IL1R2 knockout mice. **(D)** The gene expression of IL1R2 in lung tissues from IL1R2 knockout mice and control littermates was measured by qRT-PCR. Data are expressed as mean ± SEM and compared by student's t test (unpaired). *P<0.05 vs. WT.

Supplementary Figure 2

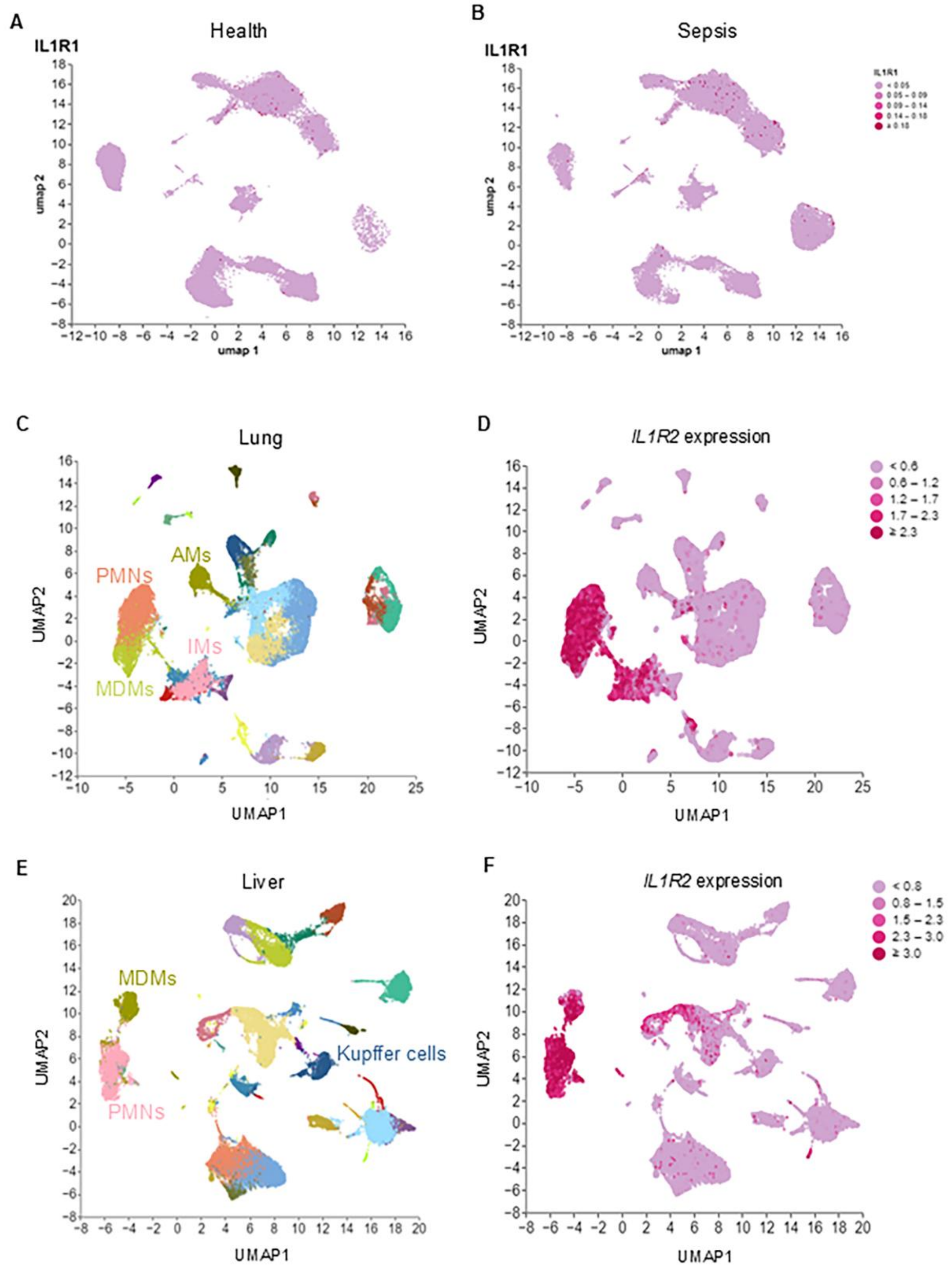

Supplementary Figure 2: *IL1R2* expression in lung and liver of septic mice. **(A, B)** UMAP representation of snRNA-seq data from blood immune cells of health control **(A)** and septic patients **(B)** colored according to IL1R1 expression. **(C)** Single-cell RNA sequencing (scRNA-seq) data from mouse lung tissues of Sham and CLP induced sepsis were analyzed and visualized using uniform manifold approximation and projection (UMAP)

plots, with colors in parentheses indicating the identified cell clusters. **(D)** UMAP representation of snRNA-seq data from lung cells colored according to *IL1R2* expression. **(E)** Single-cell RNA sequencing (scRNA-seq) data from mouse liver tissues of Sham and CLP induced sepsis were analyzed and visualized using uniform manifold approximation and projection (UMAP) plots, with colors in parentheses indicating the identified cell clusters. **(F)** UMAP representation of snRNA-seq data from liver cells colored according to *IL1R2* expression. PMNs, polymorphonuclear neutrophils; MDMs, Monocyte-derived macrophages; AMs, alveolar macrophages; IMs, interstitial macrophages.

Supplementary Figure 3

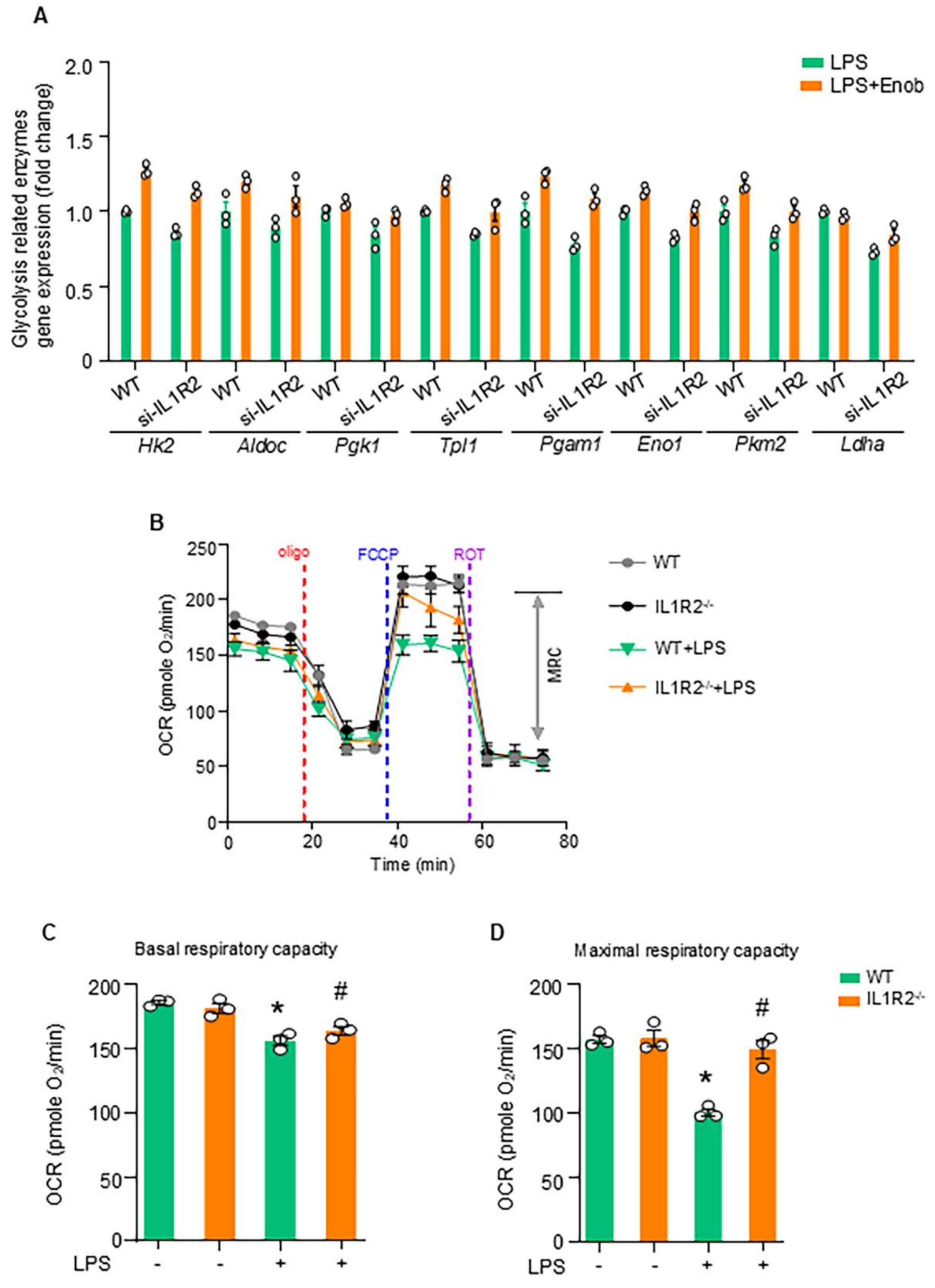

Supplementary Figure 3: Effect of IL1R2 on oxygen consumption rate (OCR) in macrophages after LPS stimulation. (A) iBMDMs were transfected with IL1R2 siRNA (si-IL1R2) or negative control (NC). After transfection the cells were treated with LPS (1 μg/ml) for 12 h. The gene expression of glycolysis related enzymes in these iBMDMs were measured byRT-PCR. **(B-D)** WT or IL1R2$^{-/-}$ primary mouse peritoneal cavity (PerC)

macrophages were stimulated with or without LPS (1μg/mL) for 12 h. The OCR in macrophages was determined by Seahorse assay. **(B)** Real-time changes in the OCR of macrophages after treatment with Oligomycin, FCCP and rotenone plus antimycin A. Baseline and maximal respiratory capacity (double-headed arrow), is shown. **(C)** Basal respiratory capacity of macrophages measured by real-time changes in OCR. **(D)** Maximal respiratory capacity of macrophages measured by real-time changes in OCR is shown. Data are expressed as mean ± SEM and compared by one-way ANOVA and Tukey's multiple comparisons test. *$p < 0.05$ vs WT+LPS (-), $^{\#}p < 0.05$ vs IL1R2$^{-/-}$ +LPS (-).

Supplementary Figure 4

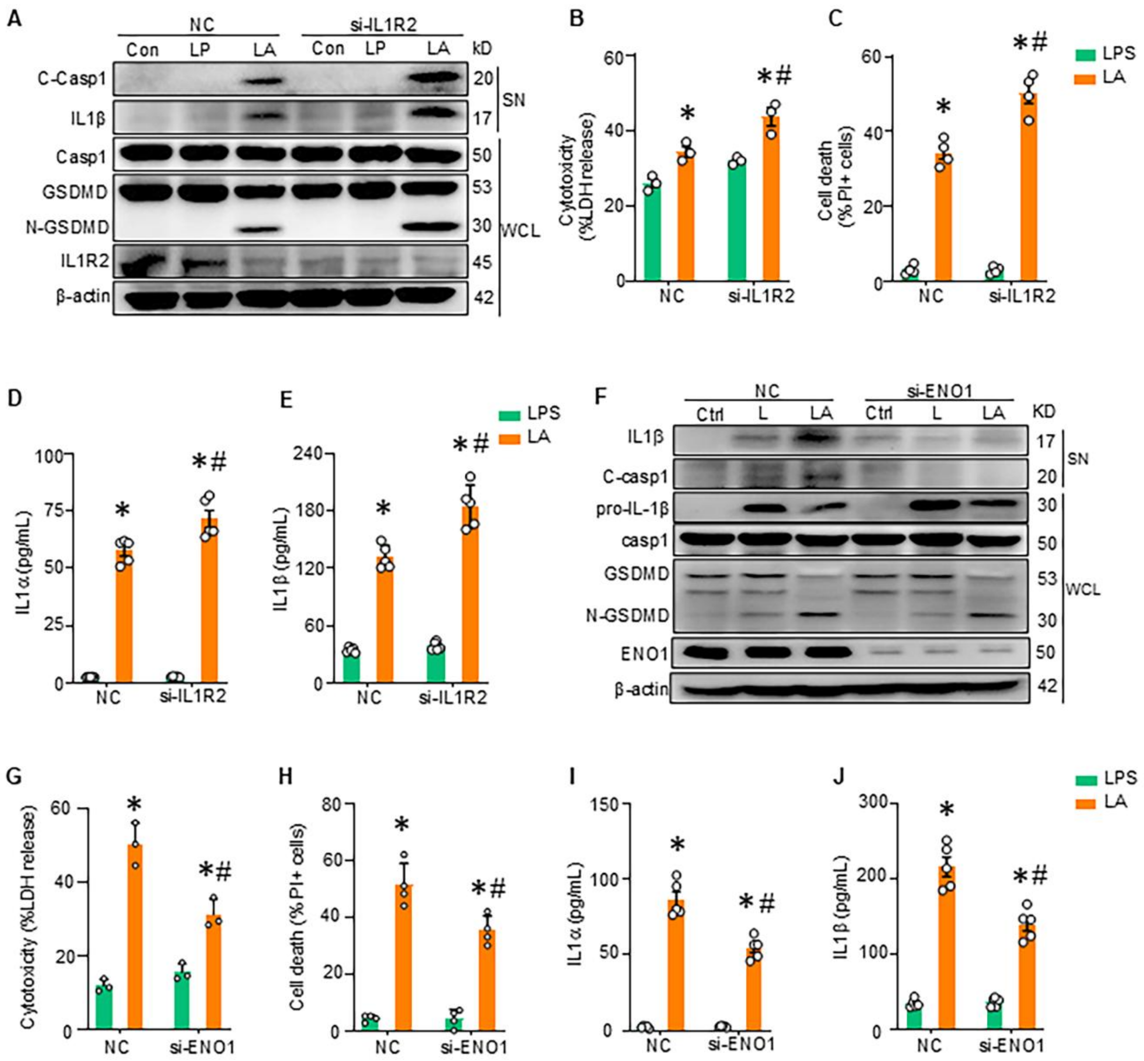

Supplementary Figure 4: The effect of IL1R2 and ENO1 on GSDMD-mediated pyroptosis. (A-E) iBMDMs were transfected with IL1R2 siRNA (si-IL1R2) or negative control (NC). After transfection the cells were treated with LPS (1 μg/ml) for 3 h, followed

by stimulation with ATP (5 mM) for 30 min. **(A)** The expressions of cleaved-caspase 1(c-casp1) and IL1β in the supernatants (SN), and the expression of casp1, GSDMD, N-GSDMD, and IL1R2 in the whole cell lysis (WCL) of these iBMDMs were measured by Western blotting. All immunoblotting experiments were repeated three times with similar results. **(B)** The levels of LDH in the culture supernatants of iBMDMs were measured by kits. **(C)** The percentage of cell death (PI^+ cells) was checked in these iBMDMs. **(D, E)** The levels of IL1α and IL1β in the culture supernatants of iBMDMs were measured by ELISA. Data are expressed as mean ± SEM and compared by two-way ANOVA and Tukey's multiple comparisons test. *$p < 0.05$ vs NC+LPS, $^{\#}p < 0.05$ vs NC+LA. **(F-J)** iBMDMs were transfected with ENO1 siRNA (si-ENO1) or negative control (NC). After transfection the cells were treated with LPS (1 μg/mL) for 3 h, followed by treated with ATP (5 mM) for 30 min. **(F)** The expression of cleaved-caspase1(c-casp1) and IL1β in the supernatants (SN), and the expression of casp1, pro-IL1β, GSDMD, N-GSDMD, and ENO1 in the whole cell lysis (WCL) of these macrophages were measured. All immunoblotting experiments were repeated three times with similar results. **(G)** The levels of LDH in the culture supernatants of macrophages were measured. **(H)** The percentage of cell death (PI^+ cells) was checked in these macrophages. **(I-J)** The levels of IL1α **(I)**, and IL1β **(J)** in the culture supernatants of macrophages were measured by ELISA kits. Data are expressed as mean ± SEM and compared by two-way ANOVA and Tukey's multiple comparisons test. *$p < 0.05$ vs NC+LPS, $^{\#}p < 0.05$ vs NC+LA. LA, LPS+ATP.

Supplementary Figure 5

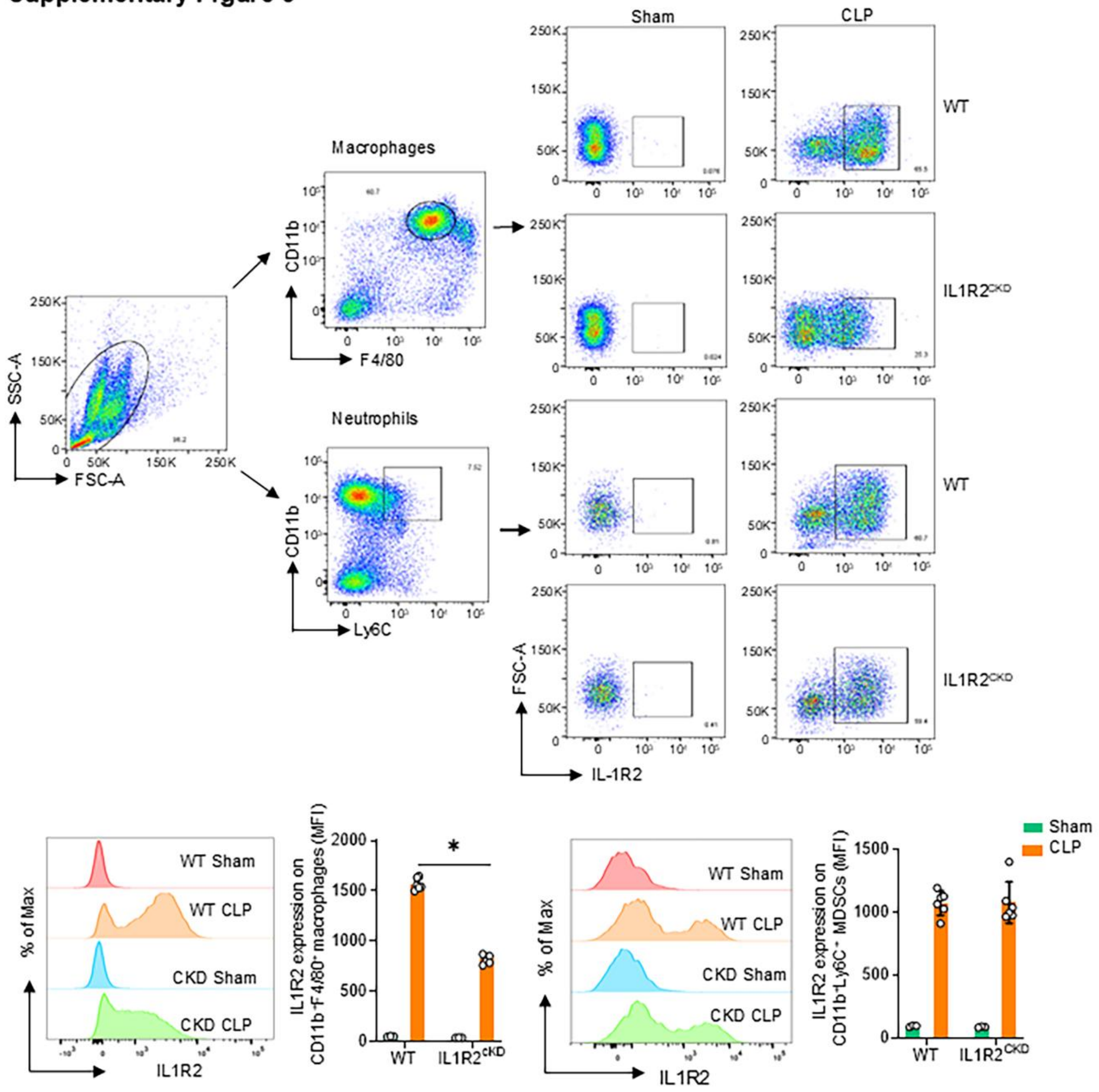

Supplementary Figure 5: Validation of generating IL1R2^{CKD} mice by Flow cytometry. The expression of IL1R2 on CD11b^{+}CD86^{+}macrophages and CD11b+Ly6G^{+} neutrophils from the bone marrow of WT and IL1R2^{CKD} mice after Sham or CLP operation were determined by flow cytometry. Data are expressed as mean ± SEM and compared by two-way ANOVA and Tukey's multiple comparisons test. *$p < 0.05$ vs indicated group.

Supplementary Figure 6

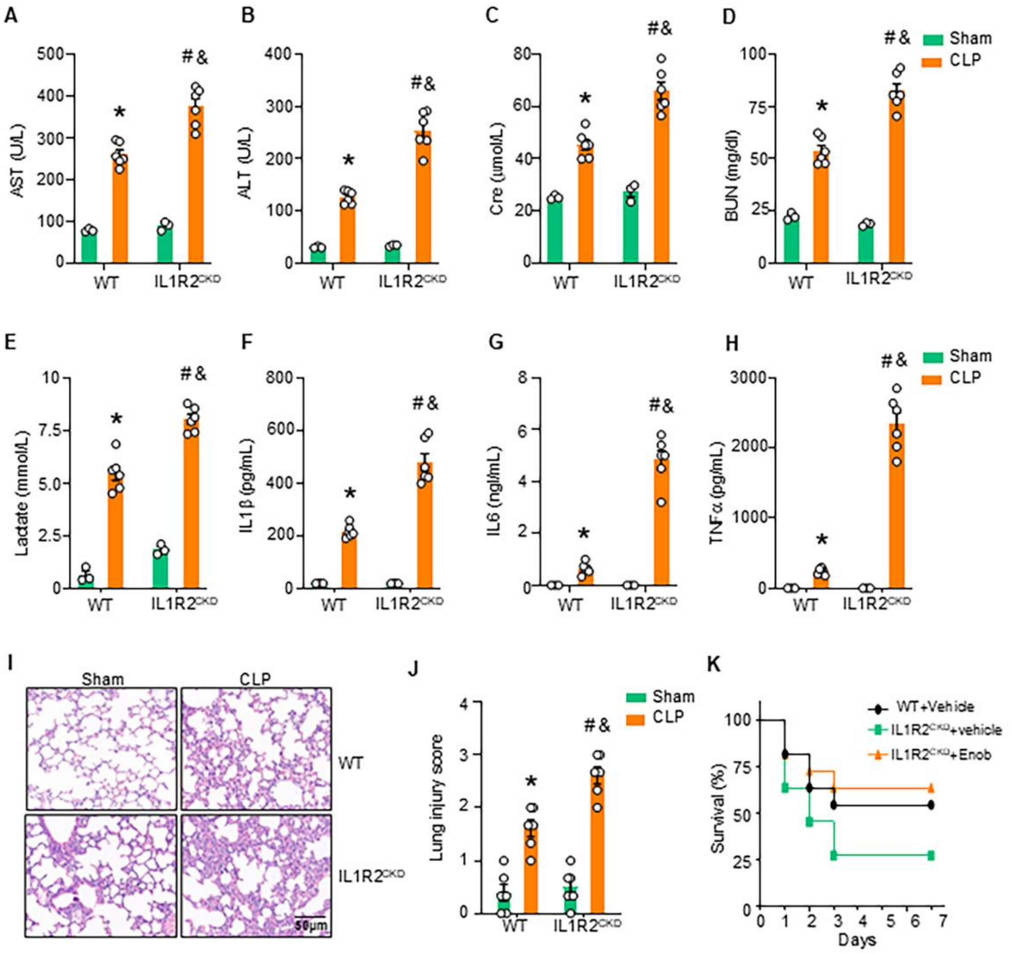

Supplementary Figure 6: Macrophage-specific IL1R2 conditioned knockout mice exhibit exacerbated organ damage in sepsis. (A-J) WT and IL1R2^{CKD} mice were assigned to sham or CLP-induced sepsis. After 20 h, blood and lung tissue were collected. **(A-D)** Plasma levels of AST **(A)**, ALT **(B)**, creatinine **(C)** and BUN **(D)** were measured by colorimetric enzymatic assay kits. **(E)** Plasma levels of lactate were measured by colorimetric assay kit. **(F-H)** Plasma levels of IL1β **(F)**, IL6 **(G)**, and TNFα **(H)** were measured by ELISA. **(I, J)** Lungs were embedded in paraffin, sectioned and analyzed for histologic injury using H&E staining and analyzed using lung injury scoring. Experiments were performed 3 times, and all data were used for analysis. Data are expressed as mean ± SEM and compared by two-way ANOVA and Tukey's multiple comparisons test. *p<0.05 vs sham WT, $^{\#}$p<0.05 vs sham IL1R2^{CKD}, $^{\&}$p<0.05 vs CLP WT. **(K)** WT and IL1R2^{CKD} mice subjected to CLP-induced sepsis with Enoblock treatment (5 mg/kg) or vehicle (volume equivalent) and monitored for 7 days for humane endpoints, and differences in survival were determined using Kaplan-Meier survival plots and a log-rank test. n= 11mice/group.

Supplementary Tables

Supplementary Table 1: Comparison of clinical characteristics and demographic data between non-sepsis vs sepsis patients

Variable	Sepsis (n = 123)	Non-sepsis (n = 29)	*P* value*
**Demographic**			
Male sex, n (%)	79 (64)	15 (52)	0.212
Age (y), M (IQR)	58 (46-68)	56 (38-72)	0.723
**Clinical history and examination on admission**			
Temperature (°C), $\bar{x} \pm s$		36.8±0.4	0.006
MAP (mmHg), $\bar{x} \pm s$	37.4±1.0	94.2±18.6	<0.001
Heart rate, $\bar{x} \pm s$	114±27	103±25	0.036
Respiratory rate, $\bar{x} \pm s$	23±6	23±6	0.908
Within MV, n (%)	106 (86)	19(66)	0.009
Duration of MV (d), M (IQR)	5(2-9)	1(0-4）	<0.001
History of antibiotic therapy, n (%)	117(95)	21(72)	<0.001
Surgery before admission, n (%)	33(27)	10(35)	0.410
**Comorbidities**			
Hypertension, n (%)	38(31)	10(35)	0.708
Diabetes, n (%)	27(22)	5(17)	0.576
Dyslipidemia, n (%)	29(24)	6(21)	0.740
Chronic lung disease, n (%)	13(10)	3(10)	1.000
Chronic liver disease, n (%)	6(5)	4(14)	0.098
Chronic renal disease, n (%)	20(16)	3(10)	0.570
Neoplasm, n (%)	22(18)	6(21)	0.726
**Infection and inflammation-associated laboratory markers**			
WBC count (10^9/L), $\bar{x} \pm s$	14.40±10.82	11.93±4.14	0.230
Neutrophils, *M(IQR)*	10.02(6.35-15.89)	10.07(8.30-13.17)	0.830
Neutrophil %, *M(IQR)*	89.9 (83.9-93.8)	89.5 (83.7-92.7)	0.808
Lymphocytes, *M(IQR)*	0.61(0.36-1.11)	0.57 (0.39-0.98)	0.576
Lymphocyte %, *M(IQR)*	5.7(3.3-9.6)	5.4 (3.0-8.3)	0.576
Monocytes, *M(IQR)*	0.36 (0.21-0.71)	0.44 (0.31-0.60)	0.386
Monocyte %, *M(IQR)*	3.3(2.0-5.3)	3.8(3.0-5.9)	0.118
Platelet count (10^{12}/L), $\bar{x} \pm s$	130±107	155±69	0.251
SOFA score, M(IQR)	11(7-14)	6(3-9)	<0.001
CRP (mg/dL), $\bar{x} \pm s$	156.14±98.47	126.64±70.15	0.385
PCT (ng/mL), $\bar{x} \pm s$	34.34±48.08	6.50±14.77	0.002
Lactate (mmol/L), $\bar{x} \pm s$	4.9±4.6	2.9±2.5	0.021
Creatinine (μmol/L), $\bar{x} \pm s$	231±247	181±245	0.325
BNP (pg/mL), $\bar{x} \pm s$	10119±10995	3843±9030	0.005
INR, *M(IQR)*	1.29(1.14-1.64)	1.17(1.05-1.34)	0.026
PT(s), $\bar{x} \pm s$	20.03 ± 19.59	15.44 ± 6.13	0.215
**Hospitalization outcomes**			
ICU stay (h), M(IQR)	179(101-313)	96(67-144)	0.001
28-days deaths, n (%)	60(49)	8(28)	0.039

Note: MAP: mean arterial pressure; MV: duration of mechanical ventilation; WBC: leukocytes; IQR: interquartile range; SOFA score: sequential assessment score of organ function; CRP:C-reactive protein; PCT: procalcitonin; BNP: B-type natriuretic peptide; INR: International Normalized Ratio; PT: prothrombin time; ICU: Intensive Care Unit. Percentages vary depending on the number of samples in the corresponding variable. *The P value was determined by the t-test when it was expressed by measurement data presented in (x) ± s and by the Mann Whitney rank-sum test when it was expressed by M(IQR). Enumeration data expressed in n (%) were compared using the $\chi 2$ test and the Fisher exact probability method was used to determine the P value.

Supplementary Table 2: Comparison of clinical characteristics and demographic data between patients with or without septic shock

Variable	With septic shock (n = 92)	Without septic shock (n = 31)	*P* value*
**Demographic**			
Male sex, n (%)	33(36)	11(35)	0.969
Age (y), *M(IQR)*	60(48-68)	54(43-69)	0.473
**Clinical history and examination on admission**			
Temperature (°C), $\bar{x} \pm s$	37(36.6-38)	37.4(36.7-37.9)	0.720
MAP (mmHg), $\bar{x} \pm s$	75(63-81)	87(76-106)	<0.001
Heart rate, $\bar{x} \pm s$	115±26	112±30	0.518
Respiratory rate, $\bar{x} \pm s$	22±6	24±7	0.249
Within MV, n (%)	83(90)	23(74)	0.025
Duration of MV (d), *M(IQR)*	5(2-10)	3(1-8)	0.110
History of antibiotic therapy, n (%)	88(96)	29(94)	0.641
Surgery before admission, n (%)	25(27)	8(26)	0.822
**Comorbidities**			
Hypertension, n (%)	18(21)	8(26)	0.653
Diabetes, n (%)	19(21)	8(26)	0.549
Dyslipidemia, n (%)	21(23)	8(26)	0.735
Chronic lung disease, n (%)	11(12)	2(6)	0.514
Chronic liver disease, n (%)	5(5)	1(3)	1.000
Chronic renal disease, n (%)	14(15)	6(19)	0.589
**Infection and inflammation-associated laboratory markers**			
WBC count (10^9/L), $\bar{x} \pm s$	15.18±11.56	12.11±7.99	0.174
Neutrophils, *M(IQR)*	10.61(7.00-16.39)	9.54(6.01-12.56)	0.195
Neutrophil %, *M(IQR)*	90.0(83.8-94.2)	89.8(84.6-92.2)	0.510
Lymphocytes, *M(IQR)*	0.65(0.38-1.12)	0.55(0.36-0.94)	0.751
Lymphocyte %, *M(IQR)*	5.6 (3.0-9.7)	6.0 (4.1-8.3)	0.484
Monocytes, *M(IQR)*	0.35(0.21-0.72)	0.37(0.23-0.64)	0.993
Monocyte %, *M(IQR)*	2.9(1.8-4.6)	3.4 (2.7-5.8)	0.120
Platelet count (10^{12}/L), $\bar{x} \pm s$	124±109	153±98	0.192
SOFA score, *M(IQR)*	12(9-14)	7(4-11)	0.010
CRP (mg/dL), $\bar{x} \pm s$	161.14±98.73	136.72±97.92	0.365
PCT (ng/mL), $\bar{x} \pm s$	39.64±51.58	18.61±31.46	0.035
Lactate (mmol/L), $\bar{x} \pm s$	5.6±4.9	3.0±2.8	0.007
Creatinine (μmol/L), $\bar{x} \pm s$	217±198	273±357	0.278
BNP (pg/mL), $\bar{x} \pm s$	11236±11495	6806±8699	0.052
INR, *M(IQR)*	1.4(1.2-1.7)	1.2(1.1-1.3)	0.009
PT(s), $\bar{x} \pm s$	21.76±22.21	14.75±3.79	0.089
D-Dimer(mg/L), $\bar{x} \pm s$	12.09±13.89	9.40±8.52	0.311
**Hospitalization outcomes**			
ICU stay (h), *M(IQR)*	157(99-293)	216(134-374)	0.087
28-days deaths, n (%)	50(54)	10(32)	0.033

Note: MAP: mean arterial pressure; MV: duration of mechanical ventilation; WBC: leukocytes; IQR: interquartile range; SOFA score: sequential assessment score of organ function; CRP:C-reactive protein; PCT: procalcitonin; BNP: B-type natriuretic peptide; INR: International Normalized Ratio; PT: prothrombin time; ICU: Intensive Care Unit. *Percentages vary depending on the number of samples in the corresponding variable. *The P value was determined by the t-test when it was expressed by measurement data presented in (x) ± s and by the Mann Whitney rank-sum test when it was expressed by M(IQR). Enumeration data expressed in n (%) were compared using the $\chi 2$ test and the Fisher exact probability method was used to determine the P value.

Supplementary Table 3: The proteins interact with IL1R2 screened by IP-MS

Accession	Protein names	Gene names	MW [kDa]	Protein score	Sequence coverage (%)	Unique Peptides	Abundances
P17182	Alpha-enolase	Eno1	47.1	219	12	3	1.10e+07
P27931	Interleukin-1 receptor type 2	Il1r2	45.6	158	8	2	6.78e+06
P21550	Beta-enolase	Eno3	47	147	8	1	1.62e+06
Q9Z130	Heterogeneous nuclear ribonucleoprotein D-like	Hnrnpdl	33.5	132	10	1	1.85e+06
P11499	Heat shock protein HSP 90-beta	Hsp90ab1	83.2	130	9	6	1.85e+07
Q64523	Histone H2A type 2-C	H2ac20	14	94	22	2	2.69e+08
P61514	60S ribosomal protein L37a	Rpl37a	10.3	88	20	1	2.08e+06
P62259	14-3-3 protein epsilon	Ywhae	29.2	84	8	2	9.90e+06
Q61233	Plastin-2	Lcp1	70.1	83	4	2	2.44e+06
Q61696	Heat shock 70 kDa protein 1A	Hspa1a	70	82	3	1	
P34884	Macrophage migration inhibitory factor	Mif	12.5	81	18	1	

Supplementary Table 4: Computational modeling of the protein-protein interaction

Complex	Surface Area ($Å^2$)	Binding ($^{\Delta i}G$) Energy (Kcal/mol)	Free Energy of Dissociation (ΔG^{diss}) (Kcal/mol)	Entropy change at dissociation ($T\Delta S^{diss}$) (Kcal/mol)	N_{HB}	N_{SB}
**IL1R2-ENO1**	3147.6	-17.3	13.0	15.0	23	3

IL1R2, interleukin 1 receptor 2; ENO1, enolase-1.

Supplementary Table 5: Primer sequences

Gene Name	Forward	Reverse
IL1R2	5'-ATCTGAATTGAGGCTGGAAGGTGAAC-3'	5'- AAACTATGGGAACTGCTGGAGATGTC-3
B-ACTIN	5'- TTCCAGCCTTCCTTCTTG-3'	5'- GGAGCCAGAGCAGTAATC-3'
IL1R2	5'-ATCTGAATTGAGGCTGGAAGGTGAAC-3'	5'- AAACTATGGGAACTGCTGGAGATGTC-3
HK2	5'-TGATCGCCTGCTTATTCACGG-3'	5'-AACCGCCTAGAAATCTCCAGA-3'
PKM2	5'-GCCGCCTGGACATTGACTC-3'	5'-CCATGAGAGAAATTCAGCCGAG-3'
PGK1	5'-ATGTCGCTTTCCAACAAGCTG-3'	5'-GCTCCATTGTCCAAGCAGAAT-3'
TPI1	5'-CCAGGAAGTTCTTCGTTGGGG-3'	5'-CAAAGTCGATGTAAGCGGTGG-3'
PGAM1	5'-TCTGTGCAGAAGAGAGCAATCC-3'	5'-CTGTCAGACCGCCATAGTGT-3'
LDHA	5'-GTCTCCAGCAAAGACTACTGT-3'	5'-GACTGTACTTGACAATGTTGGGA-3'
ALDOC	5'-AGAAGGAGTTGTCGGATATTGCT-3'	5'-TTCTCCACCCCAATTTGGCTC-3'
